# Association of *NOTCH3* Gene Polymorphisms with Ischemic Stroke and Its Subtypes: A Meta-Analysis

**DOI:** 10.3390/medicina55070351

**Published:** 2019-07-08

**Authors:** Loo Keat Wei, Lyn R. Griffiths, Irene Looi, Cheah Wee Kooi

**Affiliations:** 1 Department of Biological Science, Faculty of Science, Universiti Tunku Abdul Rahman, Bandar Barat, 31900 Kampar, Perak, Malaysia; 2 Genomics Research Centre, Institute of Health and Biomedical Innovation, Queensland University of Technology, Musk Avenue, Kelvin Grove, QLD 4059, Australia; 3 Department of Medicine and Clinical Research Centre, Seberang Jaya Hospital, Jalan Tun Hussein Onn, 13700 Seberang Jaya, Pulau Pinang, Malaysia; 4 Department of Medicine and Clinical Research Centre, Taiping Hospital, Jalan Tamingsari, 34000 Taiping, Perak, Malaysia

**Keywords:** meta-analysis, NOTCH3, single nucleotide polymorphisms (SNPs), ischemic stroke, lacunar stroke, atherothrombotic stroke

## Abstract

*Background and objectives*: *NOTCH3* gene variations play a significant role in cerebral autosomal dominant arteriopathy with subcortical infarcts and leukoencephalopathy (CADASIL). However, the role of *NOTCH3* gene polymorphisms in the risk of ischemic stroke, and its subtypes such as atherothrombotic or lacunar strokes, remains unclear. Aims: Hence, we carried out a meta-analysis to examine whether the *NOTCH3* rs1043994, rs1044009 and rs3815188 polymorphisms are associated with ischemic stroke and its major subtypes. *Materials and Methods*: All relevant studies were systematically screened and meta-analyzed using Review Manager (Revman) version 5.3. The strength of the association between *NOTCH3* polymorphisms and ischemic stroke risk and its subtypes were measured as odds ratios and 95% confidence intervals, under different genetic models. *Results*: A total of ten studies were identified, five of which considered *NOTCH3* rs1043994 (2077 cases/2147 controls), five of which considered *NOTCH3* rs1044009 (2315 cases/3053 controls), and nine of which considered *NOTCH3* rs3815188 (2819 cases/2769 controls). These studies were meta-analyzed for their association with ischemic stroke risk. Four studies (874 cases/2002 controls) of the *NOTCH3* rs3815188 polymorphism and three studies of the *NOTCH3* rs1043994 (643 cases/1552 controls) polymorphism were meta-analyzed for lacunar stroke risk. Three studies (1013 cases/1972 controls) of the *NOTCH3* rs3815188 polymorphism were meta-analyzed for atherothrombotic stroke risk. The meta-analysis results showed a lack of association between all of the studied polymorphisms and the risk of ischemic stroke and its major subtypes (i.e., atherothrombotic and lacunar). *Conclusions*: *NOTCH3* polymorphisms are not significantly associated with the risk of ischemic stroke and its subtypes (*p* < 0.05).

## 1. Introduction

Ischemic stroke is a leading cause of death and disability worldwide, especially in developing countries [1]. Ischemic stroke can be classified into five subtypes including, large-artery atherosclerosis, small vessel occlusion, cardioembolism, stroke of undetermined etiology and stroke of other determined etiology, based on the Trial of ORG 10172 in Acute Stroke Treatment (TOAST) classification. Risk factors such as age, gender, atherosclerosis, dyslipidemia, and hypertension as well as diabetes mellitus are well established [2]. However, the pathophysiological mechanism underlying ischemic stroke and its subtypes has not been fully elucidated. It is likely that both genetic and environmental factors are involved in the development of the disease [3].

The *NOTCH3* gene has been mapped to 19p13.12, spanning from 15,159,633 bp to 15,200,981 bp, and containing 33 exons and 32 introns. This gene is translated to 2321 amino acids and makes up a key protein for neuronal development [4]. NOTCH3 also regulates the survival and function of vascular smooth muscle cells, within the brain [4]. NOTCH3 contains a large extracellular domain with 34 epidermal growth factor-like repeats and an intracellular domain with seven ankyrin repeats [4]. Upon binding to a Jagged or Delta ligand, NOTCH3 is involved in three proteolytic steps before liberating the intracellular domain to the nucleus, including a proteolysis event in the trans-golgi network, a disintegrin and metalloproteinases (ADAM)-mediated proteolysis and presenilin/γ-secretase-dependent proteolysis [4]. An equal number of cysteine residues in the extracellular domain are linked by three disulphide bridges, in order to form a stable secondary epidermal growth factor-like structure [4].

*NOTCH3* rs1044009 (g.45022C>T, c.6668C>T, p.Ala2223Val) and rs3815188 (g.13568C>T, c.303C>T, p.Thr101=) polymorphisms represent a missense mutation and a synonymous variant, respectively. Both of these polymorphisms are located in the intracellular DUF3454 domain of the protein, which affects the NOTCH3 signal transduction activity directly. Nevertheless, *NOTCH3* rs1043994 (g.13949A>G, c.606A>G, p.Ala202=) is a benign point mutation or synonymous variant that is located in the conserved protein family of the calcium-binding endothelial growth factor (EGF)-like domain within the NOTCH3 protein. Mutations in the *NOTCH3* gene have been reported to be causally associated with cerebral autosomal-dominant arteriopathy with subcortical infarcts and leukoencephalopathy (CADASIL) [4,5,6,7,8].

CADASIL is an autosomal dominant cerebral small blood vessel disorder with common clinical manifestations, including migraine with aura, young to mid-age recurrent onset of ischemic stroke, apathy, psychiatric symptoms and cognitive disorder progressing to dementia [9]. Interestingly, most CADASIL patients have a demonstrated family history of stroke, in which CADASIL can increase the risk of ischemic stroke and cognitive impairment [6,10]. A study has reported that 60–85% of CADASIL patients experience their first and subsequent recurrent ischemic stroke with lacunar syndromes, aged between 45–50 years old [9]. There are 170 missense mutations known to cause CADASIL, in which 95% of them are identified in the *NOTCH3* gene [11]. Among these mutations, *NOTCH3* rs1043994, rs1044009 and rs3815188 have been associated with ischemic stroke in Chinese populations [12], but not Japanese [13,14] and Caucasian [15] populations. Indeed, the exact cause of the association of these polymorphisms with ischemic stroke risk and its subtypes remains unclear. Hence, we carried out this meta-analysis to investigate whether *NOTCH3* rs1043994, rs1044009 and rs3815188 polymorphisms are associated with ischemic stroke and its major subtypes.

## 2. Methods

### 2.1. Search Strategy

The Preferred Reporting Items for Systematic Reviews and Meta-Analyses (PRISMA) guideline was adopted in conducting this systematic review and meta-analysis. The medical subject subheading (MESH) terms that were used to identify the relevant studies were “NOTCH3”, “rs1043994”, “rs1044009”, “rs3815188”, “polymorphism”, “ischemic stroke”, “brain ischemia”, “brain infarction”, “cerebrovascular disease”, “cerebrovascular accident”, “lacunar stroke’, “small vessel disease” and “atherothrombotic stroke”. An example of a complete search algorithm was “NOTCH3 rs1043994 and ischemic stroke”. The comprehensive literature search was performed on PubMed, Scopus, Web of Science, Google scholar, WHO GHL, VHL, Jstage, KoreaMed, KSCI, POPLINE, Grey Literature Report, IMSEAR, MJM, Mycite, and WPRIM. The respective Chinese characters of these search terms were translated, in order to assess the articles deposited in the Chinese National Knowledge Infrastructure (CNKI) database. The full-text of journal articles, conference abstracts and proceedings that reported on the association between *NOTCH3* polymorphisms and ischemic stroke were downloaded from the databases [16,17]. In addition, a thorough manual searching was conducted on unpublished dissertations, theses, research reports and studies listed in the related review articles as well as secondary references listed in the publications. Literature searching was performed by two reviewers until March 2019.

### 2.2. Study Selection

The full texts of the retrieved articles were screened according to the inclusion and exclusion criteria. Studies were included if they were of case-control design, written in English or Chinese, encompassed genotypic data for odds ratios (ORs) and 95% confidence intervals (CIs) calculation. If the same group of authors has published more than one study, the most recent study with the largest sample size was incorporated into the final meta-analysis model. Qualified studies that complied with the inclusion and exclusion criteria were gathered for data extraction [18].

### 2.3. Data Extraction and Quality Assessment

Data include first author name, publication year, country of origin, ethnicity, sample size, age, genotyping method, ratio of gender, and also a definition of ischemic stroke was extracted. The genotype and allele frequencies of all cases and controls were calculated and validated. All the extracted information was compared and discussed in order to reach a consensus. The Newcastle-Ottawa Scale (NO_S_) was analyzed to examine the quality of each study. Studies with scores of six or higher were considered as high-quality studies [19].

### 2.4. Statistical Analysis

The strength of association between *NOTCH3* polymorphisms and ischemic stroke risk were estimated as OR and 95% CI under dominant, recessive, over-dominant and allelic models. Heterogeneity was enumerated using Cochran′s Q test. I^2^ values of less than 25%, 50% and 75% represented low, moderate and high heterogeneities, respectively. Based on a priori assumptions, a fixed-effect or random-effect model was chosen to evaluate the association between SNPs and ischemic stroke risk. In addition, funnel plots were inspected for publication bias [16]. All statistical tests were performed using Review Manager Version 5.3. Two-sided *p* < 0.05 were considered as statistically significant, except for P_heterogeneity_ < 0.10. In addition, a Hardy–Weinberg equilibrium (HWE) of the genotypic data was tested using chi-square.

## 3. Results

### 3.1. Studies Selection and Characteristics

**Figure 1:**
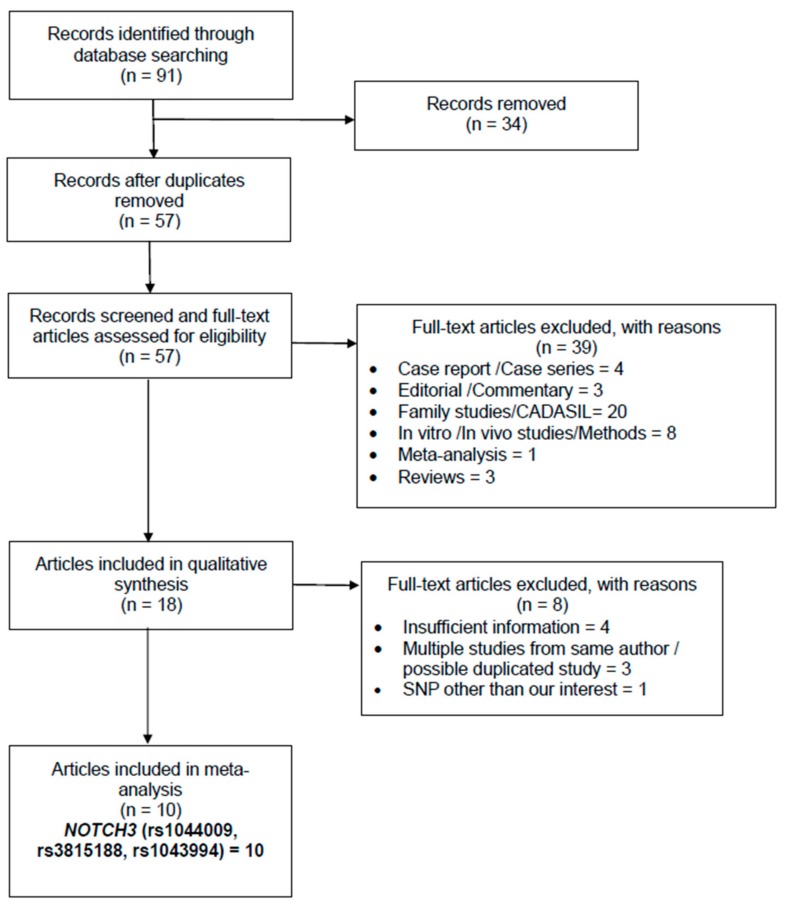
Preferred Reporting Items for Systematic Reviews and Meta-Analyses (PRISMA) flow chart of the study selection process of *NOTCH3* gene polymorphisms associated with risk of ischemic stroke.

Figure 1 demonstrated the study selection process for the association analysis between *NOTCH3* rs1043994, rs1044009 and rs3815188 polymorphisms and ischemic stroke risk. Ninety-one articles retrieved from the initial database search, were screened for duplicates (n = 34), case reports (n = 4), editorials and commentaries (n = 3), family studies (n = 20), in vitro or in vivo studies (n = 8), meta-analysis (n = 1), reviews (n = 3), and the remaining articles (n = 18) for secondary screening. A total of eight articles were excluded in the secondary screening due to insufficient information (n = 4), possible duplicated study from the same team of authors (n = 3) and SNPs other than ones of interest (n = 1). Finally, a total of ten articles were identified, five of which considered *NOTCH3* rs1043994 (2077 cases/2147 controls), five of which considered *NOTCH3* rs1044009 (2315 cases/3053 controls), and nine of which considered *NOTCH3* rs3815188 (2819 cases/2769 controls). These articles were meta-analyzed for their association with ischemic stroke risk (Figure 1). As for the lacunar stroke, four articles (874 cases/2002 controls) of *NOTCH3* rs3815188 polymorphism and three articles (643 cases/1552 controls) of *NOTCH3* rs1043994 polymorphism were meta-analyzed. Three articles consisting of 1013 cases and 1972 controls were meta-analyzed for the association between *NOTCH3* rs3815188 polymorphism and atherothrombotic stroke risk.

The main characteristics of all included studies are summarized in Table 1. All the included studies were of Asian populations, except for Wang et al. [20] and Ross et al. [15]. Forty percent of the studies were matched for age and gender [12,14,20,21], while 10% of the studies were matched for age and demographic area [22]. Among the included studies, the control group reported by Yuan et al. [12] consisted of both population- and hospital-based controls; 80% of the cases were assessed by neuroimaging techniques except for Zhu et al. [23], by a stroke specialist. In addition, 30% of the included studies deviated from HWE (*p* < 0.05) [13,22,24]. For the NOS score, all the included studies scored 7–8 points, except for Ghoreishizadeh et al. [25], Li et al. [24] and Mizuno et al. [13] scoring only 6 points due to a lack of information.

### 3.2. Quantitative Synthesis and Subgroup Analysis

As shown in Table 2, 83.3% of ischemic stroke outcomes were derived from fixed-effect models. Meanwhile, dominant and allelic models of rs3815188 were random-effect models. The pooled meta-analysis indicated that *NOTCH3* rs1043994, rs1044009 and rs3815188 polymorphisms were not associated with ischemic stroke under any genetic model (Table 2, Supplementary Figures S1–S3).

With regard to ischemic stroke subtypes, the overall outcomes of lacunar and atherothrombotic stroke were analyzed under fixed-effect models. In contrast, dominant and allelic models of the *NOTCH3* rs3815188 polymorphism with lacunar stroke were investigated under random-effect models. Dominant and over-dominant models of *NOTCH3* rs3815188 polymorphism in atherothrombotic stroke were interpreted from random-effect models as well. Notably, none of the genetic models of *NOTCH3* rs1043994 and rs3815188 polymorphisms were significantly associated with lacunar stroke (Table 3, Supplementary Figures S4 and S5). Additionally, the *NOTCH3* rs3815188 polymorphism was not significantly associated with atherothrombotic stroke, under any genetic model (Table 4 and Supplementary Figure S6).

A minimum of three studies is generally needed to ensure the validity of conclusions drawn by a meta-analysis. Hence, subgroup analysis stratified by populations was not undertaken for the association of *NOTCH3* rs1043994, rs3815188 and rs1044009 polymorphisms with ischemic stroke and its subtypes, due to less than three studies reported in non-Asian populations in most of the genetic models.

### 3.3. Heterogeneity and Publication Bias

The results of Cochran’s Q test and I^2^ values indicated a moderate heterogeneity in the dominant and allelic models of rs3815188 polymorphism in ischemic stroke (Table 2, Supplementary Figures S2 and S3). Higher levels of heterogeneity were observed in the dominant and allelic models of *NOTCH3* rs3815188 polymorphism among lacunar stroke (Table 3, Supplementary Figure S5). Similarly, a moderate level of heterogeneity was detected in the dominant and over-dominant models of *NOTCH3* rs3815188 polymorphism among atherothrombotic stroke Table 4, Supplementary Figure S6. In addition, a normal distribution of funnel plot was found for all the genetic models, indicating that no publication bias was detected in this meta-analysis.

## 4. Discussion

This comprehensive meta-analysis investigated the potential association of (1) *NOTCH3* rs1043994, rs1044009 and rs3815188 polymorphisms with ischemic stroke risk; (2) *NOTCH3* rs1043994 and rs3815188 polymorphisms with lacunar stroke risk; and (3) *NOTCH3* rs3815188 polymorphism with atherothrombotic stroke risk. All the studied polymorphisms were not significantly associated with the risk of ischemic stroke and its subtypes. These findings are consistent with the report by González-Giraldo et al. [26], suggesting a non-significant association between *NOTCH3* polymorphisms and ischemic stroke risk. However, their meta-analysis only included two polymorphisms of *NOTCH3* (i.e., rs1043994 and rs3815188) with a relatively smaller number of studies (i.e., Wang et al. [22], Mizuno et al. [13], Ross et al. [15]). Moreover, their results were limited to the allelic models of *NOTCH3* rs1043994 and rs3815188 polymorphisms, and no information was available on ischemic stroke subtypes [26]. In addition, the ultimate goals of this meta-analysis involved (1) investigating the potential association of *NOTCH3* rs1044009 polymorphism and ischemic stroke; (2) examining the possible relationship between these three polymorphisms and the major stroke subtypes; and (3) covering all the available studies (10 studies, with 7211 cases 7969 controls).

Ross et al. [15] reported on the association results between SNPs and ischemic stroke risks for two different ethnicities, who were recruited from the Ischemic Stroke Genetics Study, with study subjects from Caucasian and African American descents, treated as separate studies in the subsequent analysis. Univariate and multivariate analyses demonstrated that despite *NOTCH3* rs3515188, rs1043994 and rs1044009 polymorphisms exerting susceptibility risks towards and/or protective risks against, ischemic stroke in both ethnicities, they were not statistically significant [15]. The author argued that Type II error is an unavoidable issue in their study, particularly after performing multiple testing adjustments on the African American series [15].

Mizuno et al. [13] suggested that the *NOTCH3* rs3515188 polymorphism may not be exerting a significant pathological role for sporadic stroke because of the positive natural selection of the *NOTCH3* rs3515188 T allele amongst the Japanese population. Similarly, Ito et al. [14] failed to determine a significant association between the *NOTCH3* rs1044009 polymorphism and cerebrovascular disease risk in a Japanese population. The author suggested the non-significant association may be due to the fact that most of the cases showed positive findings on leukoencephalopathy, a predominant characteristic for CADASIL [14]. Li et al. [24] observed that the *NOTCH3* rs1044009 AG genotype was more abundant amongst lacunar stroke patients with leukoaraiosis compared to healthy controls, as well as patients with pure lacunar stroke. Thus, suggesting that the *NOTCH3* rs1044009 polymorphism may be linked to CADASIL or lacunar stroke but not ischemic stroke in general [14]. Future studies that determine whether the *NOTCH3* rs1044009 polymorphism affects ischemic stroke risk or lacunar stroke risk using a prospective cohort study design rather than using a case-control design is warranted [14]. Our meta-analysis was currently unable to tackle this because we were unable to obtain more than two prospective cohort studies with adequate genotypic data published on either ischemic stroke risk or lacunar stroke risk, however such studies should be considered when more data is available in future.

It is inevitable that demographic profiles such as age, gender, and ethnicity can affect SNP-disease risk at a population level. Thus, the demographic characteristics of matched case-control studies (Liu et al. [22], Wang et al. [20], Xu et al. [21] and Yuan et al. [12]) are often preferred over unmatched studies (Li et al. [24] and Zhu et al. [23]). Whilst Liu et al. [22], Xu et al. [21] and Yuan et al. [12] demonstrated a relatively similar age group for both cases and controls, Wang et al. [20] failed to follow the trend. Wang et al. [20] conducted an age-matched case-control study, with young stroke defined as patients who were aged between 20 to 90 years old. Thus, the mean ages for cases and controls were 68.8 and 60.1 years old, respectively [20]. Large differences in age groups become more obvious when an unmatched case control study such as Li et al. [24] was reported. For example, the mean age gaps between cases and controls reported by Li et al. [24] were up to 10 years old (i.e., 68.9 and 58.9 for cases and controls, respectively).

NOTCH3 signaling may exert a beneficial effect on the pathogenesis of ischemic stroke, by protecting against vascular smooth muscle cells apoptosis [27]. Loss of NOTCH3 function has been found to increase susceptibility to cortical spreading depression, causing migraine with aura also associated with ischemic stroke [28]. Under an ischemic condition, the wave of cortical spreading depression from the peri infarct area is able to perfuse peri-ischemic tissue repeatedly. This in turn, contributes to an increased metabolic demand on ischemic penumbra and enhances the size of the ischemic core [28]. It has been revealed that *NOTCH3* polymorphisms can maintain the function and integrity of blood vessels, thus, preventing individuals from lacunar stroke and possibly athrothrombotic stroke [14,24]. In contrast, benign polymorphisms reported in this meta-analysis, i.e., *NOTCH3* rs1043994, rs3515188 and rs1044009, were unable to exert any gain-of function or loss-of function on the NOTCH3 protein [14] and this may explain why these *NOTCH3* polymorphisms were not associated with ischemic stroke risk in this meta-analysis.

Another possible reason for the observed non-significant association may be due to the fact that none of these studied polymorphisms are participating in disulphide bridge formation. It has been reported that odd numbers of cysteine residues in the epidermal growth factor-like domain may impair the formation of disulphide bridges [29]. Abnormal disulphide bridge formation can lead to the misfolding of the epidermal growth factor-like domain, subsequently enhancing the extracellular domain NOTCH3 multimerization [29]. However, whether or not cysteine-sparing mutations are correlated with ischemic stroke remains debatable, although excessive accumulation and deposition of NOTCH3 in the small resistance arteries may cause arteriopathy, and thus reduce cerebral blood flow during the development of ischemic stroke [29].

A few limitations were unavoidable in the current meta-analysis. The sample size reported in this meta-analysis was relatively small, since only ten clinical studies have been undertaken on these associations. Therefore, the included studies of *NOTCH3* rs1043994, rs1044009 and rs3815188 polymorphisms could not be stratified into Asian and non-Asian populations. Moreover, different genotyping methods may contribute to HWE deviations and between-study heterogeneity. Despite these limitations, this is the most comprehensive meta-analysis that provides solid evidence for the non-significant association of *NOTCH3* polymorphisms with ischemic stroke and its major subtypes (i.e., atherothrombotic and lacunar).

## 5. Conclusions

Based on the meta-analysis data, we may conclude that *NOTCH3* polymorphisms are not significantly associated with the risk of ischemic stroke and its subtypes.

## Figures and Tables

**Table 1:** Main characteristic of studies incorporated in the final meta-analysis model.

				Total Number			Mean Age (Years)		Male							
Author	Year	Country	Ethnicity	Case	Control	Matching	Case	Control	Case	Control	Source of Controls	Clinical Diagnosis	Neuroimaging Method	Genotyping Method	SNP(s)	NOS
Ghoreishizadeh, A. [25]	2018	Iran	A	65	65	-	52 ± 14.3	54 ± 11.7	0.51	0.57	HB	-	-	PCR-SSCP	rs3815188	6
Ito, D. [14]	2002	Japan	A	235	315	A&S	<71	-	-	-	HB	-	CT/MRI	PCR-RFLP	rs1044009	8
Li, Y. [24]	2013	China	A	276	118	-	68.9 ± 10.9	58.9 ± 10.2	0.5	0.5	HB	-	CT/MRI	PCR-sequencing	rs3815188, rs1043994	6
Liu, J. [22]	2009	China	A	1823	1832	A&D	60.3 ± 9.4	59.6 ± 8.8	0.6	0.6	HB	-	CT/MRI	PCR-RFLP	rs1044009 ^§^, rs3815188 ^§^, rs1043994 ^§^	7
Mizuno, T. [13]	2002	Japan	A	111	23	-	70.4 ± 10.9 *	64.7 ± 11.6	0.5 *	0.5	-	-	MRI/MRA	PCR-dHPLC	rs3815188 ^§^, rs1043994	6
Ross, O. [15]	2013a	USA	C	452	350	-	-	-	-	-	-	-	CT/MRI	Taqman assay	rs1044009, rs3815188, rs1043994	7
Ross, O. [15]	2013b	USA	African	167	131	-	-	-	-	-	-	-	CT/MRI	Taqman assay	rs1044009, rs3815188, rs1043994	7
Wang, T. [20]	2000	UK	C	70	117	A&S	68.8 ± 13.6	60.1 ± 15.0	0.6	0.4	-	-	Radiological	PCR-RFLP	rs3815188, rs1043994	7
Xu, Y. [21]	2015	China	A	445	200	A&S	64.2 ± 12.2	62.6 ± 12.1	0.6	0.6	HB	-	CT/MRI	PCR-RFLP	rs3815188	8
Yuan, X. [12]	2016	China	A	134	115	A&S	59.3 ± 9.0	61.5 ± 10.2	0.6	0.6	HB, PB	-	-	PCR-sequencing	rs3815188	7
Zhu, C. [23]	2016	China	A	260	600	-	64.9 ± 11.7	41.4 ± 6.9 **, 66.1 ± 4.2 ***	0.7	0.7	HB	Specialist	MRI	PCR-sequencing	rs1044009 ^§^	6

A: Asian, A&D, Age and demographic area, A&S, Age and Sex, C: Caucasian, CT: Computer tomography, HB: hospital-based, NOS: Newcastle-Ottawa Scale, PCR-dHPLC: Polymerase chain reaction-denatured high performance liquid chromatography, PCR-RFLP: Polymerase chain reaction-restriction fragment length polymorphism; PCR-SSCP: Polymerase chain reaction-single strand conformation polymorphism; MRA: Magnetic resonance angiography, MRI: Magnetic resonance imaging, PB: population-based, TC: total cholesterol, UK: United Kingdom, USA: United State of America. Age and Male for cases and controls were rounding off to the nearest significant figures. Age was indicated as mean ± standard deviation. * data were based on lacunar stroke alone, ** controls of younger age group, and *** controls of older age group. - indicates missing data. ^§^ Hardy Weinberg Equilibrium. ^a^ and ^b^ are from the same study, Ross O et al., (2013).

**Table 2:** The association between *NOTCH3* genetic polymorphisms and ischemic stroke.

	Models			
	Dominant	Recessive	Over-Dominant	Allelic
rs1043994	1.09 [0.93–1.28], 0.31	1.08 [0.67–1.75], 0.74	0.90 [0.76–1.07], 0.23	0.96 [0.83–1.10], 0.54
	I^2^ = 0%; 0.96	I^2^ = 0%; 0.57	I^2^ = 0%; 0.89	I^2^ = 0%; 0.89
rs1044009	0.94 [0.84–1.06], 0.32	1.16 [0.97–1.38], 0.10	1.00 [0.88–1.12], 0.96	1.12 [0.98–1.17], 0.11
	I^2^ = 0%; 0.66	I^2^ = 33%; 0.20	I^2^ = 30%; 0.22	I^2^ = 0%; 0.80
rs3815188	0.89 [0.70–1.14], 0.36 *	1.07 [0.93–1.23], 0.35	1.03 [0.92–1.16], 0.57	1.10 [0.93–1.30], 0.25 *
	I^2^ = 59% *p* = 0.02	I^2^ = 18%, 0.29	I^2^ = 42%, 0.10	I^2^ = 52% *p* = 0.03

* Odds Ratio (OR) was calculated by random effects model. NS: not significant. OR [95% CI], *p* value.

**Table 3:** The association between *NOTCH3* genetic polymorphisms and lacunar stroke.

	Models			
	Dominant	Recessive	Over-Dominant	Allelic
rs1043994	1.17 [0.90–1.51], 0.24	1.48 [0.74–2.96], 0.27	0.79 [0.60–1.04], 0.09	0.92 [0.73–1.17], 0.50
	I^2^ = 0%; 0.88	I^2^ = 0%; 0.84	I^2^ = 0%; 0.82	I^2^ = 0%; 0.81
rs3815188	1.04 [0.64–1.67], 0.89 *	1.04 [0.86–1.26], 0.66	0.95 [0.80–1.13], 0.55	1.05 [0.76–1.45], 0.77 *
	I^2^ = 71%; 0.02	I^2^ = 35%; 0.20	I^2^ = 42%; 0.16	I^2^ = 70%; 0.02

OR [95% CI], *p* value. * OR was calculated by random effects model. NS: not significant.

**Table 4:** The association between *NOTCH3* genetic polymorphisms and atherothrombotic stroke.

	Models			
	Dominant	Recessive	Over-Dominant	Allelic
rs3815188	1.06 [0.69–1.63], 0.80 *	1.08 [0.91–1.28], 0.39	0.93 [0.66–1.32], 0.89 *	1.06 [0.95–1.18], 0.31
	I^2^ = 63%; 0.07	I^2^ = 0%; 0.75	I^2^ = 53%; 0.12	I^2^ = 28%; 0.25

OR [95% CI], *p* value. * OR was calculated by random effects model. NS: not significant.

## Supplementary Materials

The following are available online at https://www.mdpi.com/1010-660X/55/7/351/s1, Figure S1: The association between rs1043994 and ischemic stroke risk, under (a) dominant, (b) recessive, (c) over-dominant and (d) allelic models, Figure S2: The association between rs1044009 and ischemic stroke risk, under (a) dominant, (b) recessive, (c) over-dominant and (d) allelic models, Figure S3: The association between rs3815188 and ischemic stroke risk, under (a) dominant, (b) recessive (c) over-dominant and (d) allelic models, Figure S4: The association between rs1043994 and lacunar stroke risk, under (a) dominant, (b) recessive, (c) over-dominant and (d) allelic models, Figure S5: The association between rs3815188 and lacunar stroke risk, under (a) dominant, (b) recessive, (c) over-dominant and (d) allelic models, Figure S6: The association between rs3815188 and atherothrombotic stroke risk, under (a) dominant, (b) recessive, (c) over-dominant and (d) allelic models, Figure S7: Funnel plot of rs1043994 and ischemic stroke risk, under (a) dominant, (b) recessive, (c) over-dominant and (d) allelic models, Figure S8: Funnel plot of rs1044009 and ischemic stroke risk, under (a) dominant, (b) recessive, (c) over-dominant and (d) allelic models, Figure S9: Funnel plot of rs3815188 and ischemic stroke risk, under (a) dominant, (b) recessive (c) over-dominant and (d) allelic models, Figure S10: Funnel plot of rs1043994 and lacunar stroke risk, under (a) dominant, (b) recessive, (c) over-dominant and (d) allelic models, Figure S11: Funnel plot of rs3815188 and lacunar stroke risk, under (a) dominant, (b) recessive, (c) over-dominant and (d) allelic models, Figure S12: Funnel plot of rss3815188 and atherothrombotic stroke risk, under (a) dominant, (b) recessive, (c) over-dominant and (d) allelic models, Table S1: Supplementary Table 1 Genotype of *NOTCH3* rs1043994, rs1044009 and rs3815188 polymorphisms.
