# Supplementary material for: Association of NOTCH3 Gene Polymorphisms with Ischemic Stroke and Its Subtypes: A Meta-Analysis
Source: Medicina (Kaunas). 2019 Jul 8;55(7):351. doi: 10.3390/medicina55070351 (PMC6681102; doi:10.3390/medicina55070351)
Supplement: Supplementary file 1 [file medicina-55-00351-s001.zip › Supplementary Figures.pdf]

a)

Study or Subgroup	Weight	Odds Ratio M–H, Fixed, 95% CI
Mizuno, T 2002	1.8%	0.83 [0.22, 3.10]
Ross, O 2013a	24.5%	0.99 [0.71, 1.39]
Li, Y 2013	3.4%	1.02 [0.42, 2.48]
Liu, J 2009	63.4%	1.12 [0.92, 1.37]
Ross, O 2013b	7.0%	1.17 [0.65, 2.12]
**Total (95% CI)**	**100.0%**	**1.09 [0.93, 1.28]**
Total events		

Heterogeneity: $Chi^2 = 0.62$, df = 4 (P = 0.96); $I^2 = 0\%$
Test for overall effect: Z = 1.01 (P = 0.31)

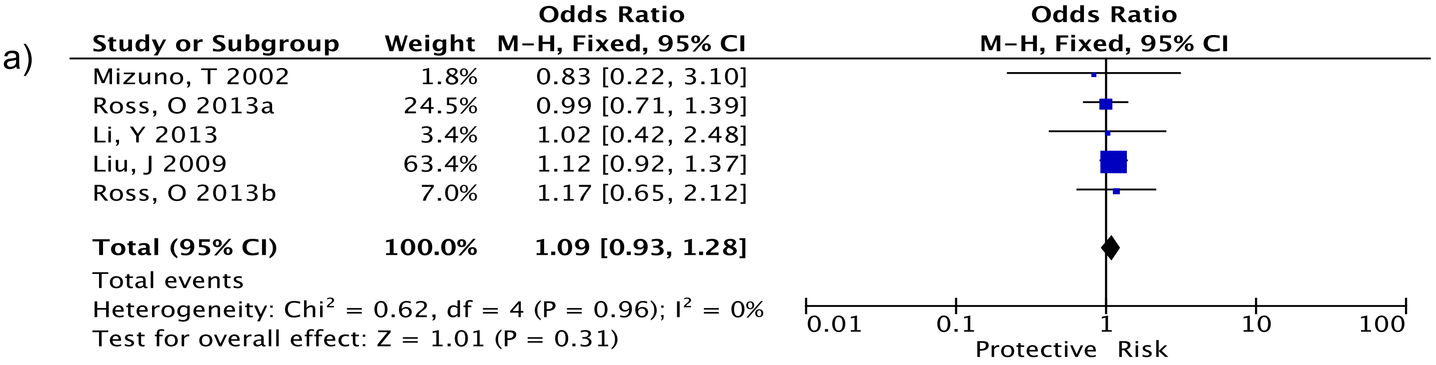

b)

Study or Subgroup	Weight	Odds Ratio M–H, Fixed, 95% CI
Ross, O 2013b	10.3%	0.26 [0.03, 2.50]
Mizuno, T 2002	2.5%	0.64 [0.03, 16.15]
Ross, O 2013a	23.9%	0.77 [0.27, 2.21]
Liu, J 2009	61.0%	1.28 [0.72, 2.30]
Li, Y 2013	2.3%	3.34 [0.18, 62.10]
**Total (95% CI)**	**100.0%**	**1.08 [0.67, 1.75]**
Total events		

Heterogeneity: $Chi^2 = 2.93$, df = 4 (P = 0.57); $I^2 = 0\%$
Test for overall effect: Z = 0.33 (P = 0.74)

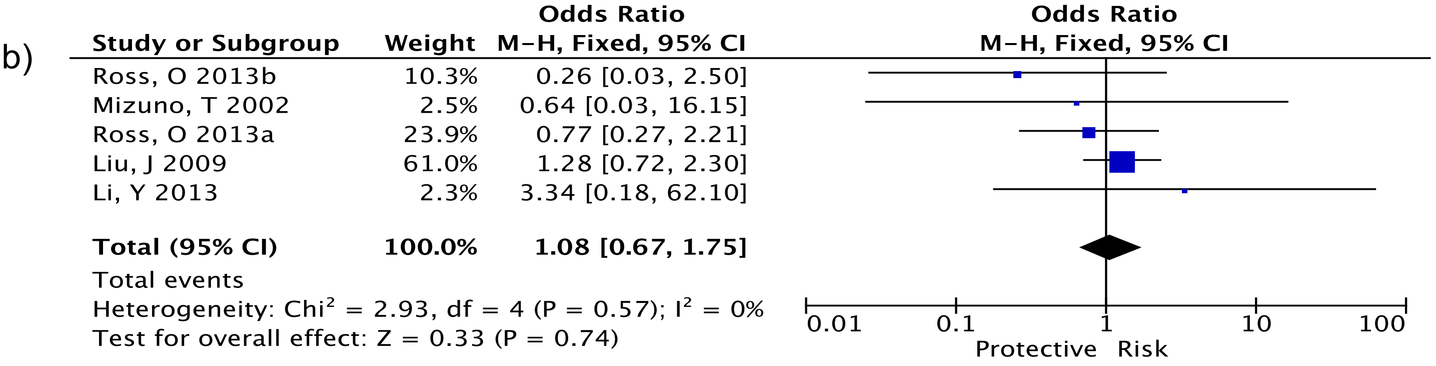

c)

Study or Subgroup	Weight	Odds Ratio M–H, Fixed, 95% CI
Li, Y 2013	3.6%	0.77 [0.31, 1.88]
Liu, J 2009	65.6%	0.85 [0.69, 1.05]
Ross, O 2013b	7.1%	0.96 [0.52, 1.77]
Ross, O 2013a	22.2%	1.03 [0.73, 1.46]
Mizuno, T 2002	1.5%	1.12 [0.30, 4.22]
**Total (95% CI)**	**100.0%**	**0.90 [0.76, 1.07]**
Total events		

Heterogeneity: $Chi^2 = 1.14$, df = 4 (P = 0.89); $I^2 = 0\%$
Test for overall effect: Z = 1.21 (P = 0.23)

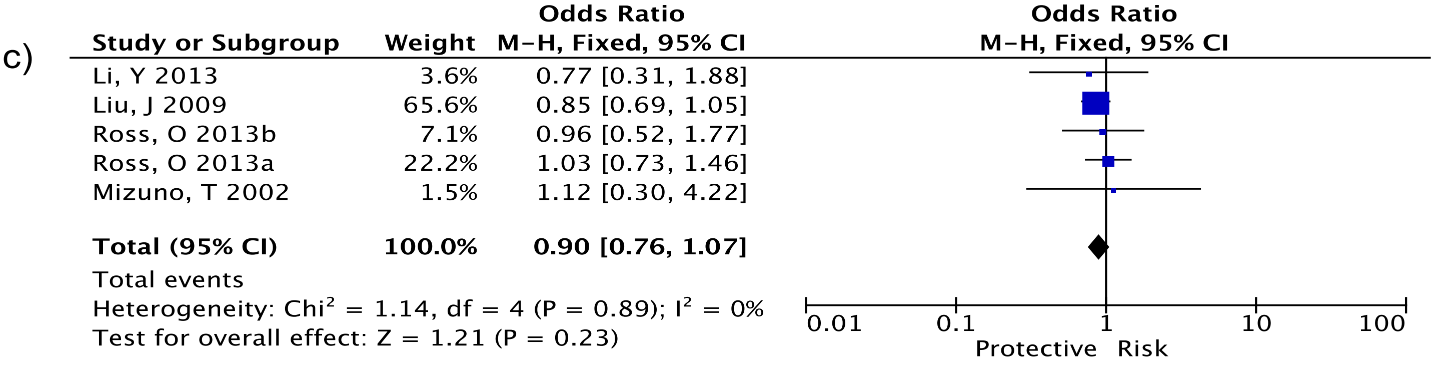

d)

Study or Subgroup	Weight	Odds Ratio M–H, Fixed, 95% CI
Ross, O 2013b	7.4%	0.79 [0.46, 1.37]
Liu, J 2009	62.1%	0.93 [0.77, 1.12]
Ross, O 2013a	22.3%	0.98 [0.73, 1.33]
Li, Y 2013	3.0%	1.17 [0.53, 2.59]
Mizuno, T 2002	1.2%	1.26 [0.36, 4.48]
Wang, T 2000	3.9%	1.27 [0.65, 2.47]
**Total (95% CI)**	**100.0%**	**0.96 [0.83, 1.10]**
Total events		

Heterogeneity: $Chi^2 = 1.70$, df = 5 (P = 0.89); $I^2 = 0\%$
Test for overall effect: Z = 0.61 (P = 0.54)

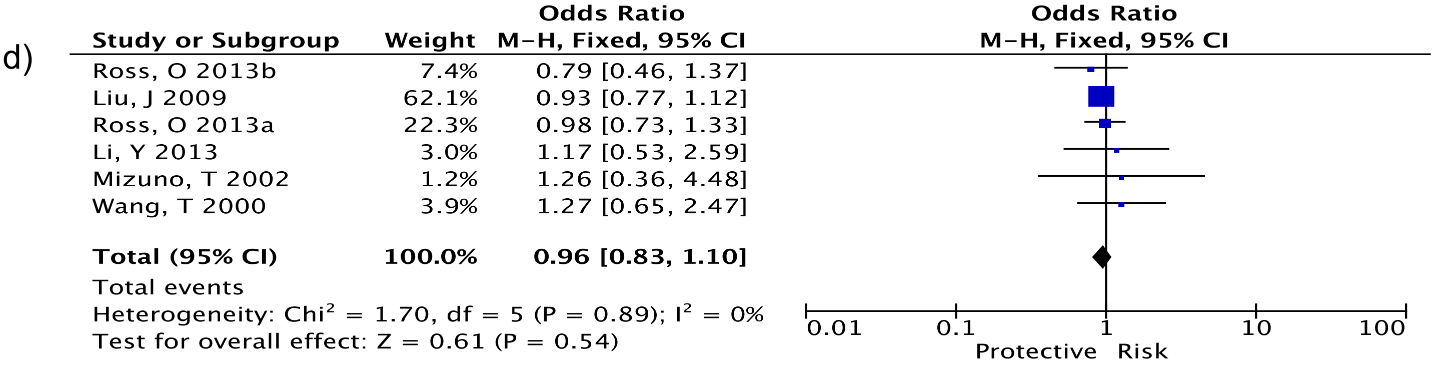

Supplementary Figure 1: The association between rs1043994 and ischemic stroke risk, under (a) dominant, (b) recessive, (c) over-dominant and (d) allelic models.

a)

Study or Subgroup	Weight	Odds Ratio M–H, Fixed, 95% CI
Zhu, C 2016	15.5%	0.82 [0.61, 1.11]
Ross, O 2013a	14.5%	0.87 [0.64, 1.19]
Liu, J 2009	56.7%	0.95 [0.82, 1.11]
Ross, O 2013b	4.0%	1.05 [0.60, 1.84]
Ito, D 2002	9.3%	1.16 [0.81, 1.65]
**Total (95% CI)**	**100.0%**	**0.94 [0.84, 1.06]**
Total events		

Heterogeneity: $Chi^2 = 2.43$, df = 4 (P = 0.66); $I^2 = 0\%$
Test for overall effect: Z = 1.00 (P = 0.32)

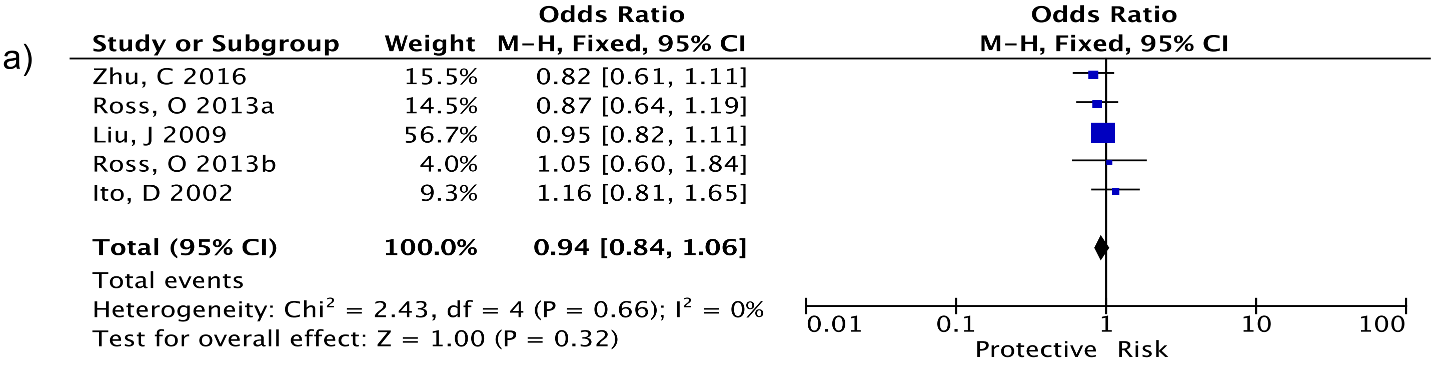

b)

Study or Subgroup	Experimental Events	Experimental Total	Control Events	Control Total	Weight	Odds Ratio M-H, Fixed, 95% CI
Ross, O 2013a	14	417	16	294	8.0%	0.60 [0.29, 1.26]
Zhu, C 2016	54	260	125	600	26.4%	1.00 [0.70, 1.43]
Ito, D 2002	31	211	45	315	13.6%	1.03 [0.63, 1.69]
Ross, O 2013b	35	154	19	95	8.0%	1.18 [0.63, 2.21]
Liu, J 2009	130	1273	132	1749	44.0%	1.39 [1.08, 1.80]
**Total (95% CI)**		**2315**		**3053**	**100.0%**	**1.16 [0.97, 1.38]**
Total events	264		337			

Heterogeneity: $Chi^2 = 5.96$, df = 4 (P = 0.20); $I^2 = 33\%$
Test for overall effect: Z = 1.64 (P = 0.10)

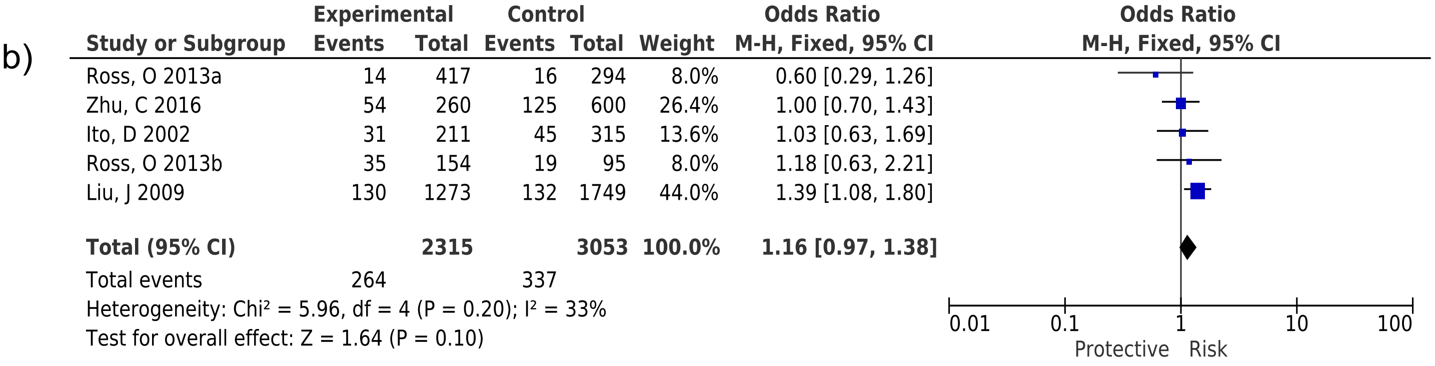

c)

Study or Subgroup	Experimental Events	Experimental Total	Control Events	Control Total	Weight	Odds Ratio M-H, Fixed, 95% CI
Ito, D 2002	93	211	151	315	12.5%	0.86 [0.60, 1.22]
Ross, O 2013b	72	154	48	95	5.8%	0.86 [0.52, 1.43]
Liu, J 2009	321	1273	468	1749	54.3%	0.92 [0.78, 1.09]
Zhu, C 2016	113	260	233	600	14.7%	1.21 [0.90, 1.63]
Ross, O 2013a	157	417	95	294	12.8%	1.26 [0.92, 1.73]
**Total (95% CI)**		**2315**		**3053**	**100.0%**	**1.00 [0.88, 1.12]**
Total events	756		995			

Heterogeneity: $Chi^2 = 5.75$, df = 4 (P = 0.22); $I^2 = 30\%$
Test for overall effect: Z = 0.05 (P = 0.96)

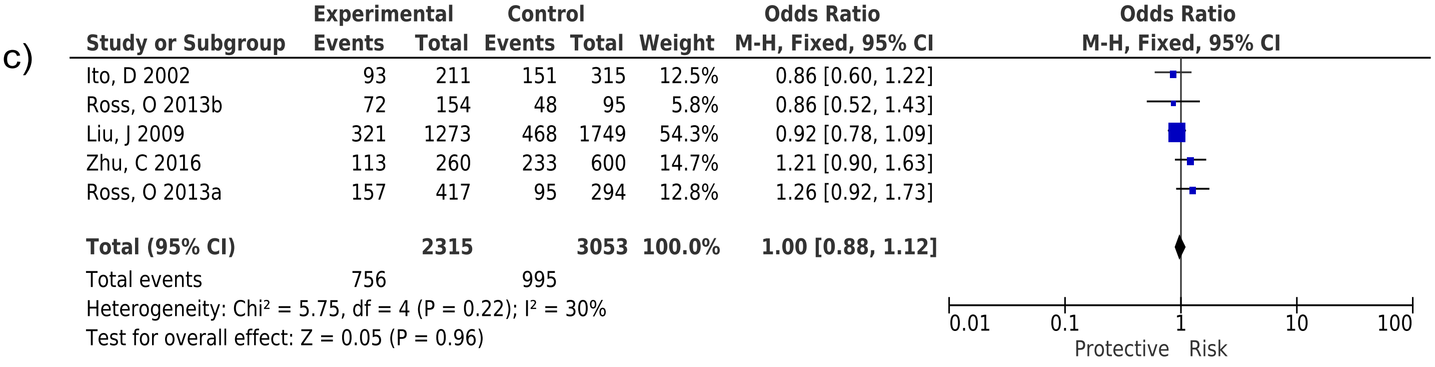

d)

Study or Subgroup	Experimental Events	Experimental Total	Control Events	Control Total	Weight	Odds Ratio M-H, Fixed, 95% CI
Ito, D 2002	155	422	241	630	13.0%	0.94 [0.73, 1.21]
Ross, O 2013b	142	308	86	190	6.1%	1.03 [0.72, 1.49]
Ross, O 2013a	185	834	127	588	12.3%	1.03 [0.80, 1.34]
Zhu, C 2016	221	520	483	1200	17.9%	1.10 [0.89, 1.35]
Liu, J 2009	581	2546	732	3498	50.7%	1.12 [0.99, 1.26]
**Total (95% CI)**		**4630**		**6106**	**100.0%**	**1.07 [0.98, 1.17]**
Total events	1284		1669			

Heterogeneity: $Chi^2 = 1.66$, df = 4 (P = 0.80); $I^2 = 0\%$
Test for overall effect: Z = 1.60 (P = 0.11)

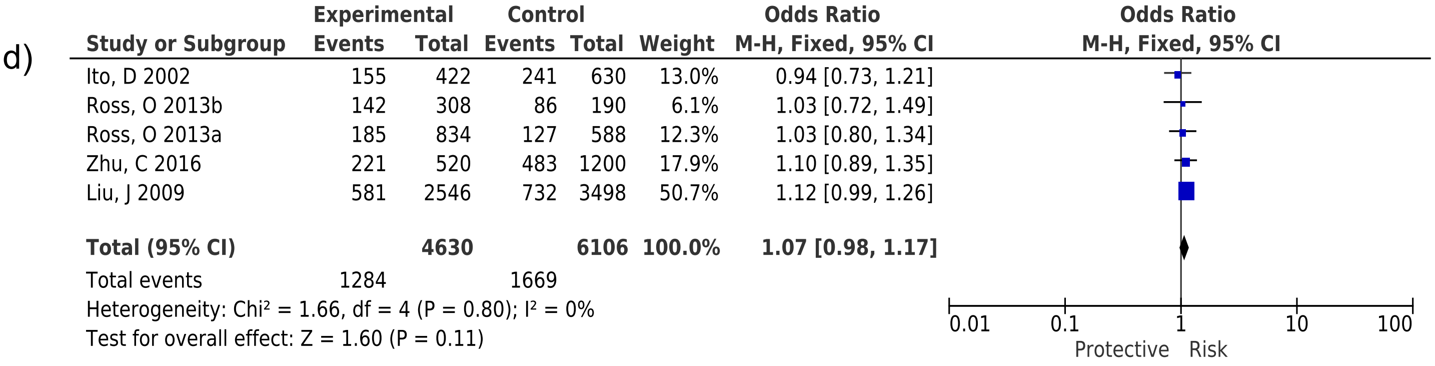

Supplementary Figure 2: The association between rs1044009 and ischemic stroke risk, under (a) dominant, (b) recessive, (c) over-dominant and (d) allelic models.

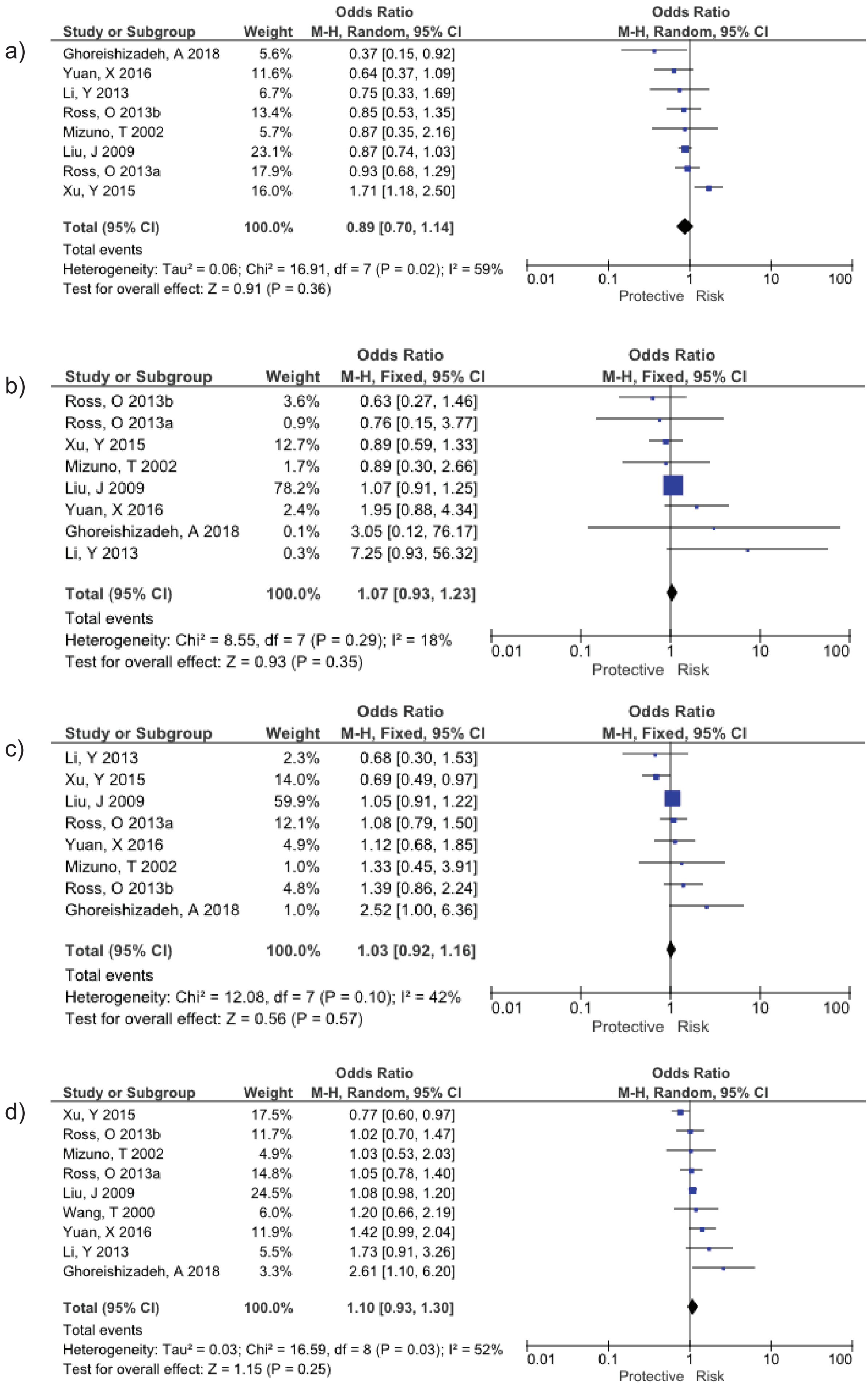

Supplementary Figure 3: The association between rs3815188 and ischemic stroke risk, under (a) dominant, (b) recessive (c) over-dominant and (d) allelic models.

a)

Study or Subgroup	Weight	Odds Ratio M-H, Fixed, 95% CI
Li, Y 2013	8.9%	1.02 [0.42, 2.48]
Liu, J 2009	88.9%	1.17 [0.89, 1.54]
Mizuno,T 2002	2.3%	1.62 [0.35, 7.41]
**Total (95% CI)**	**100.0%**	**1.17 [0.90, 1.51]**
Total events		

Heterogeneity: $Chi^2 = 0.26$, df = 2 (P = 0.88); $I^2 = 0\%$
Test for overall effect: Z = 1.17 (P = 0.24)

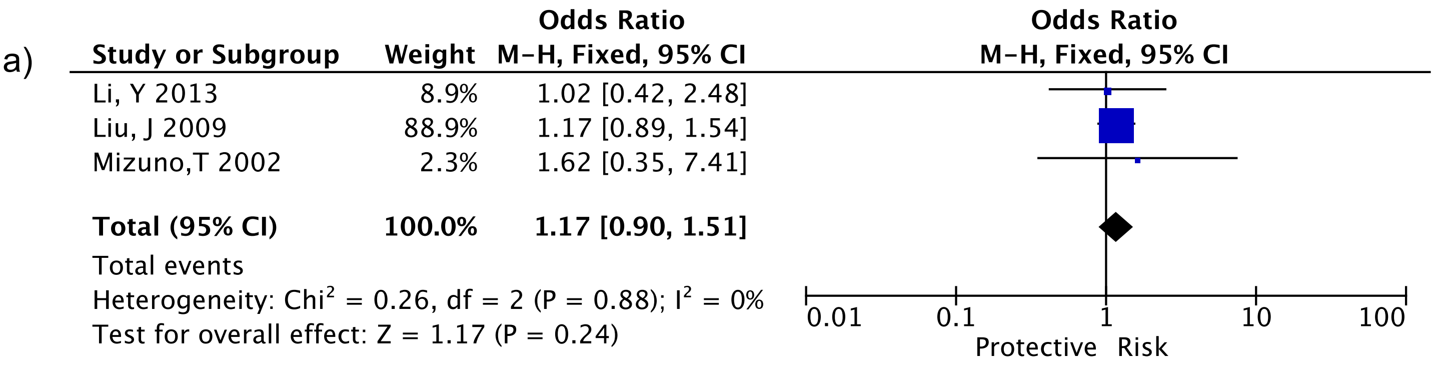

b)

Study or Subgroup	Weight	Odds Ratio M-H, Fixed, 95% CI
Mizuno,T 2002	5.6%	1.21 [0.05, 30.65]
Liu, J 2009	88.4%	1.37 [0.65, 2.90]
Li, Y 2013	5.9%	3.34 [0.18, 62.10]
**Total (95% CI)**	**100.0%**	**1.48 [0.74, 2.96]**
Total events		

Heterogeneity: $Chi^2 = 0.35$, df = 2 (P = 0.84); $I^2 = 0\%$
Test for overall effect: Z = 1.10 (P = 0.27)

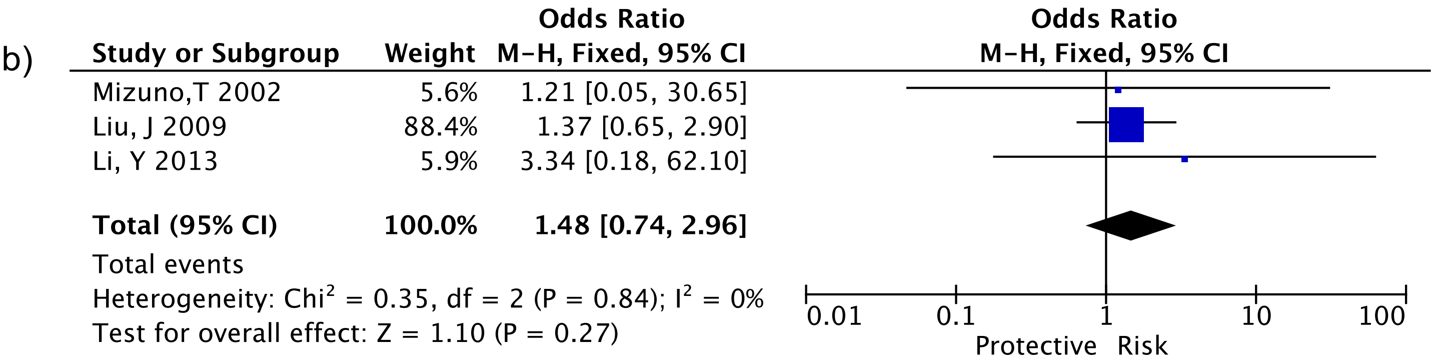

c)

Study or Subgroup	Weight	Odds Ratio M-H, Fixed, 95% CI
Mizuno,T 2002	3.3%	0.48 [0.10, 2.36]
Li, Y 2013	8.7%	0.77 [0.31, 1.88]
Liu, J 2009	87.9%	0.81 [0.60, 1.08]
**Total (95% CI)**	**100.0%**	**0.79 [0.60, 1.04]**
Total events		

Heterogeneity: $Chi^2 = 0.39$, df = 2 (P = 0.82); $I^2 = 0\%$
Test for overall effect: Z = 1.67 (P = 0.09)

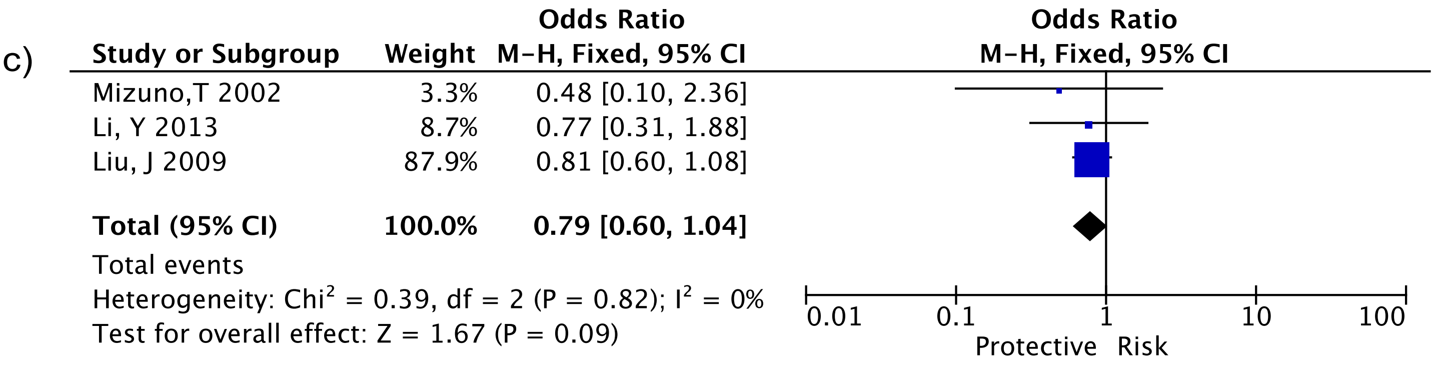

d)

Study or Subgroup	Weight	Odds Ratio M-H, Fixed, 95% CI
Mizuno,T 2002	2.8%	0.77 [0.18, 3.21]
Liu, J 2009	89.4%	0.91 [0.71, 1.16]
Li, Y 2013	7.8%	1.17 [0.53, 2.59]
**Total (95% CI)**	**100.0%**	**0.92 [0.73, 1.17]**
Total events		

Heterogeneity: $Chi^2 = 0.43$, df = 2 (P = 0.81); $I^2 = 0\%$
Test for overall effect: Z = 0.67 (P = 0.50)

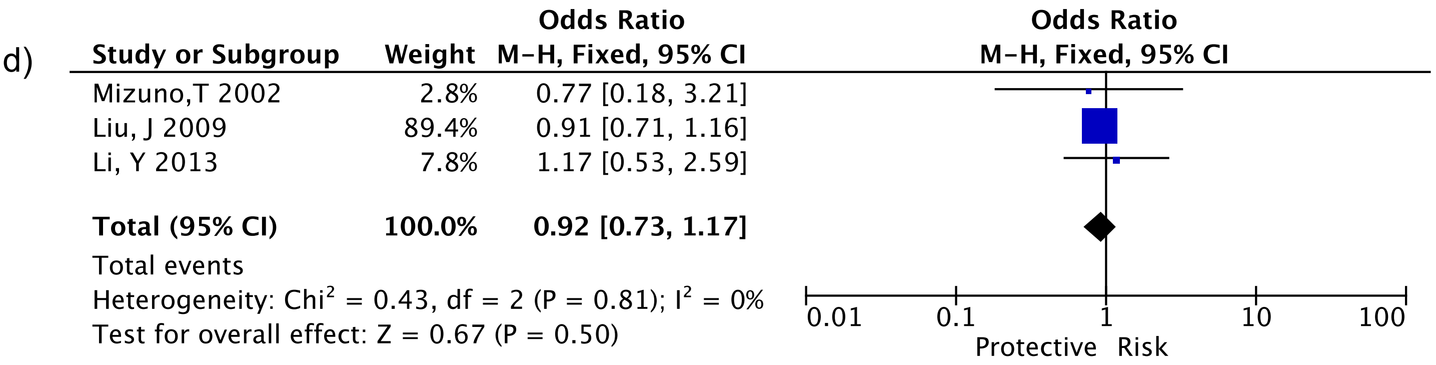

Supplementary Figure 4: The association between rs1043994 and lacunar stroke risk, under (a) dominant, (b) recessive, (c) over-dominant and (d) allelic models.

a)

Study or Subgroup	Weight	Odds Ratio M-H, Random, 95% CI
Mizuno, T 2002	15.1%	0.65 [0.25, 1.71]
Li, Y 2013	18.5%	0.75 [0.33, 1.69]
Liu, J 2009	36.3%	0.90 [0.72, 1.13]
Xu, Y 2015	30.0%	1.88 [1.23, 2.89]
**Total (95% CI)**	**100.0%**	**1.04 [0.64, 1.67]**
Total events		

Heterogeneity: $Tau^2 = 0.15$; $Chi^2 = 10.33$, df = 3 (P = 0.02); $I^2 = 71\%$
Test for overall effect: Z = 0.14 (P = 0.89)

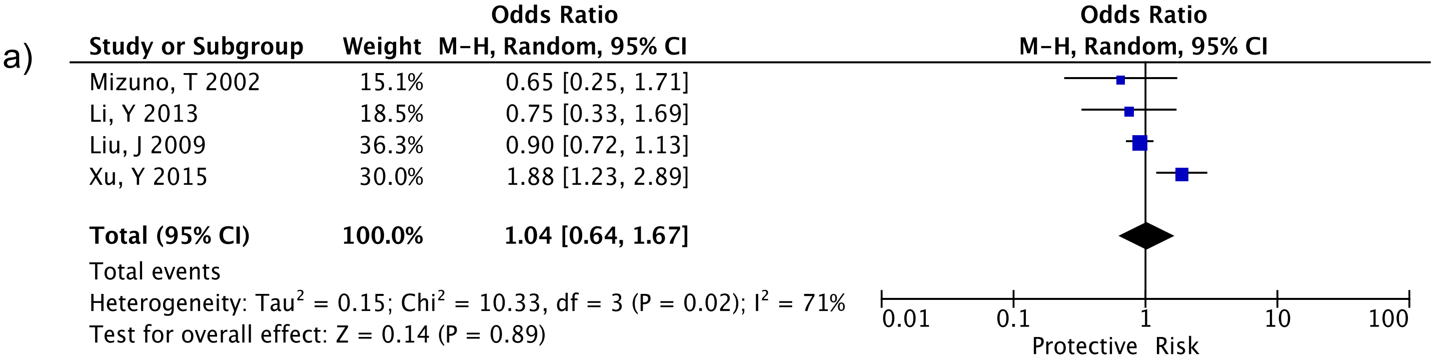

b)

Study or Subgroup	Weight	Odds Ratio M-H, Fixed, 95% CI
Xu, Y 2015	17.7%	0.82 [0.50, 1.33]
Liu, J 2009	79.1%	1.04 [0.84, 1.29]
Mizuno, T 2002	2.6%	1.34 [0.43, 4.21]
Li, Y 2013	0.6%	7.25 [0.93, 56.32]
**Total (95% CI)**	**100.0%**	**1.04 [0.86, 1.26]**
Total events		

Heterogeneity: $Chi^2 = 4.60$, df = 3 (P = 0.20); $I^2 = 35\%$
Test for overall effect: Z = 0.44 (P = 0.66)

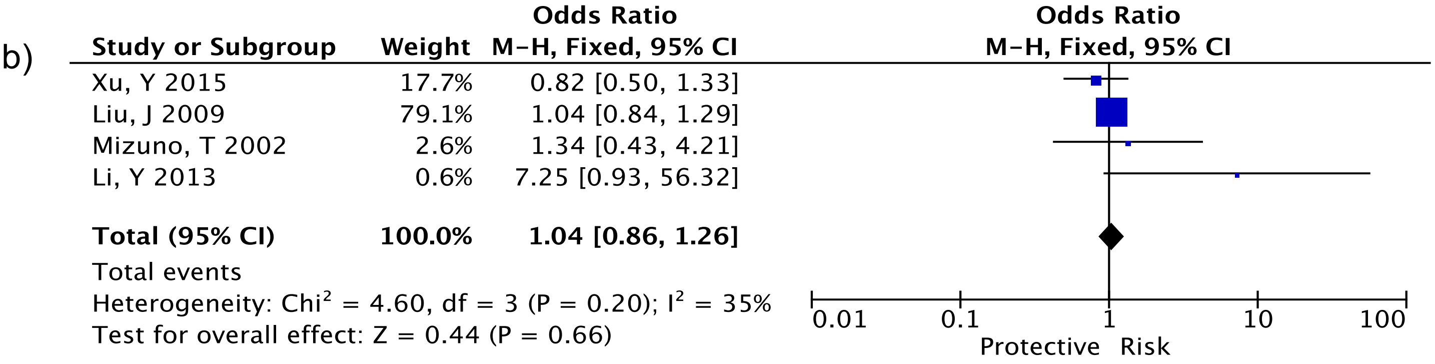

c)

Study or Subgroup	Weight	Odds Ratio M-H, Fixed, 95% CI
Xu, Y 2015	22.8%	0.67 [0.45, 0.99]
Li, Y 2013	5.1%	0.68 [0.30, 1.53]
Liu, J 2009	70.2%	1.05 [0.86, 1.28]
Mizuno, T 2002	2.0%	1.34 [0.43, 4.21]
**Total (95% CI)**	**100.0%**	**0.95 [0.80, 1.13]**
Total events		

Heterogeneity: $Chi^2 = 5.16$, df = 3 (P = 0.16); $I^2 = 42\%$
Test for overall effect: Z = 0.59 (P = 0.55)

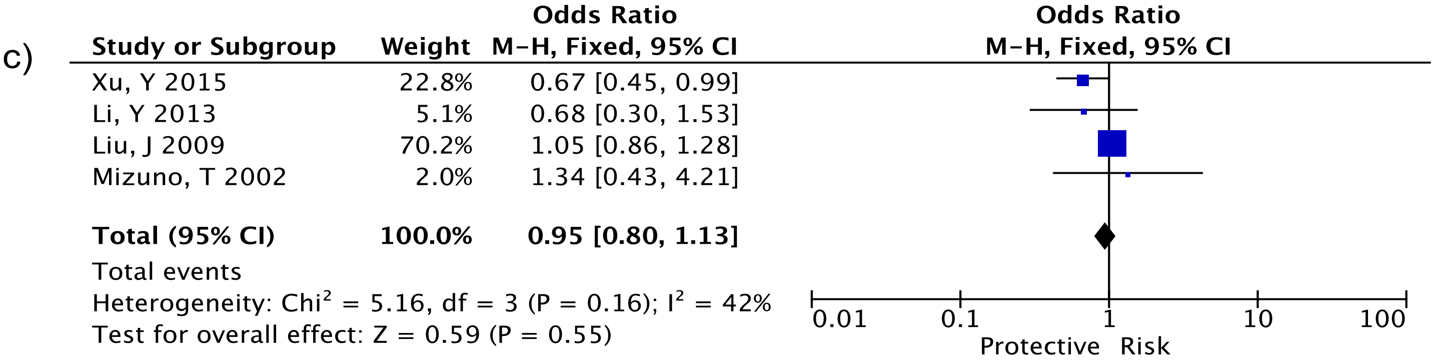

d)

Study or Subgroup	Weight	Odds Ratio M-H, Random, 95% CI
Xu, Y 2015	31.8%	0.71 [0.54, 0.94]
Liu, J 2009	38.5%	1.06 [0.92, 1.22]
Mizuno, T 2002	13.7%	1.42 [0.69, 2.90]
Li, Y 2013	16.0%	1.73 [0.91, 3.26]
**Total (95% CI)**	**100.0%**	**1.05 [0.76, 1.45]**
Total events		

Heterogeneity: $Tau^2 = 0.07$; $Chi^2 = 10.00$, df = 3 (P = 0.02); $I^2 = 70\%$
Test for overall effect: Z = 0.29 (P = 0.77)

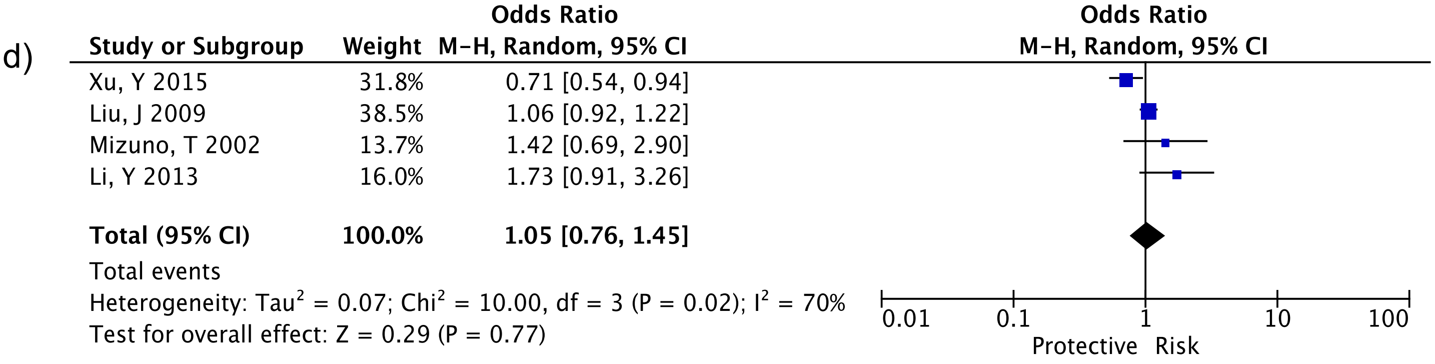

Supplementary Figure 5: The association between rs3815188 and lacunar stroke risk, under (a) dominant, (b) recessive, (c) over-dominant and (d) allelic models.

a)

Study or Subgroup	Weight	Odds Ratio M-H, Random, 95% CI	Odds Ratio M-H, Random, 95% CI
Liu, J 2009	50.9%	0.85 [0.70, 1.04]	
Mizuno, T 2002	12.7%	0.92 [0.32, 2.70]	
Xu, Y 2015	36.4%	1.50 [0.97, 2.30]	
**Total (95% CI)**	**100.0%**	**1.06 [0.69, 1.63]**	
Total events			

Heterogeneity: $Tau^2 = 0.09$; $Chi^2 = 5.45$, df = 2 (P = 0.07); $I^2 = 63\%$
Test for overall effect: Z = 0.25 (P = 0.80)

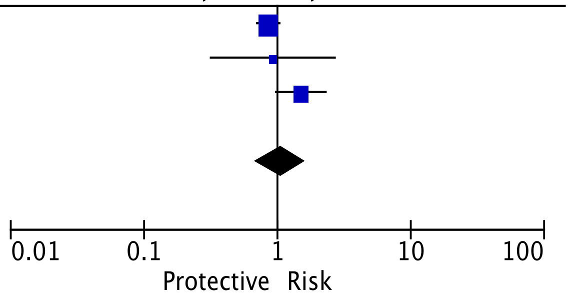

b)

Study or Subgroup	Weight	Odds Ratio M-H, Fixed, 95% CI	Odds Ratio M-H, Fixed, 95% CI
Mizuno, T 2002	2.0%	0.64 [0.16, 2.54]	
Xu, Y 2015	13.8%	1.05 [0.66, 1.67]	
Liu, J 2009	84.3%	1.09 [0.91, 1.31]	
**Total (95% CI)**	**100.0%**	**1.08 [0.91, 1.28]**	
Total events			

Heterogeneity: $Chi^2 = 0.57$, df = 2 (P = 0.75); $I^2 = 0\%$
Test for overall effect: Z = 0.86 (P = 0.39)

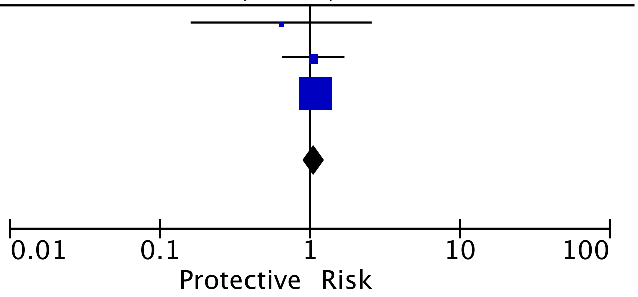

c)

Study or Subgroup	Weight	Odds Ratio M-H, Fixed, 95% CI	Odds Ratio M-H, Fixed, 95% CI
Xu, Y 2015	19.2%	0.69 [0.47, 1.02]	
Liu, J 2009	79.5%	1.05 [0.89, 1.25]	
Mizuno, T 2002	1.3%	1.57 [0.45, 5.40]	
**Total (95% CI)**	**100.0%**	**0.99 [0.85, 1.16]**	
Total events			

Heterogeneity: $Chi^2 = 4.23$, df = 2 (P = 0.12); $I^2 = 53\%$
Test for overall effect: Z = 0.14 (P = 0.89)

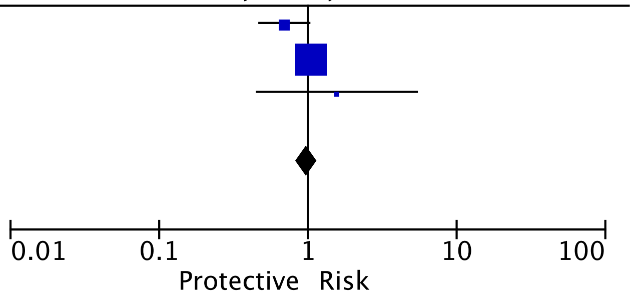

d)

Study or Subgroup	Weight	Odds Ratio M-H, Fixed, 95% CI	Odds Ratio M-H, Fixed, 95% CI
Xu, Y 2015	17.6%	0.86 [0.66, 1.13]	
Mizuno, T 2002	2.0%	0.90 [0.40, 2.02]	
Liu, J 2009	80.4%	1.10 [0.98, 1.24]	
**Total (95% CI)**	**100.0%**	**1.06 [0.95, 1.18]**	
Total events			

Heterogeneity: $Chi^2 = 2.76$, df = 2 (P = 0.25); $I^2 = 28\%$
Test for overall effect: Z = 1.01 (P = 0.31)

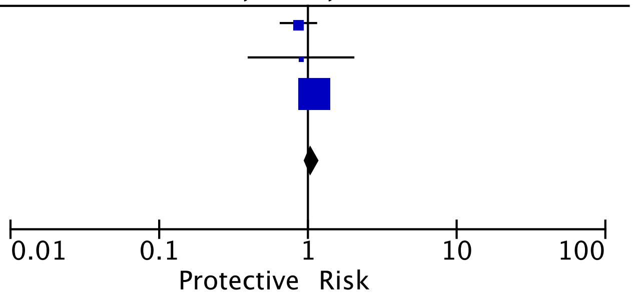

Supplementary Figure 6: The association between rs3815188 and atherothrombotic stroke risk, under (a) dominant, (b) recessive, (c) over-dominant and (d) allelic models.

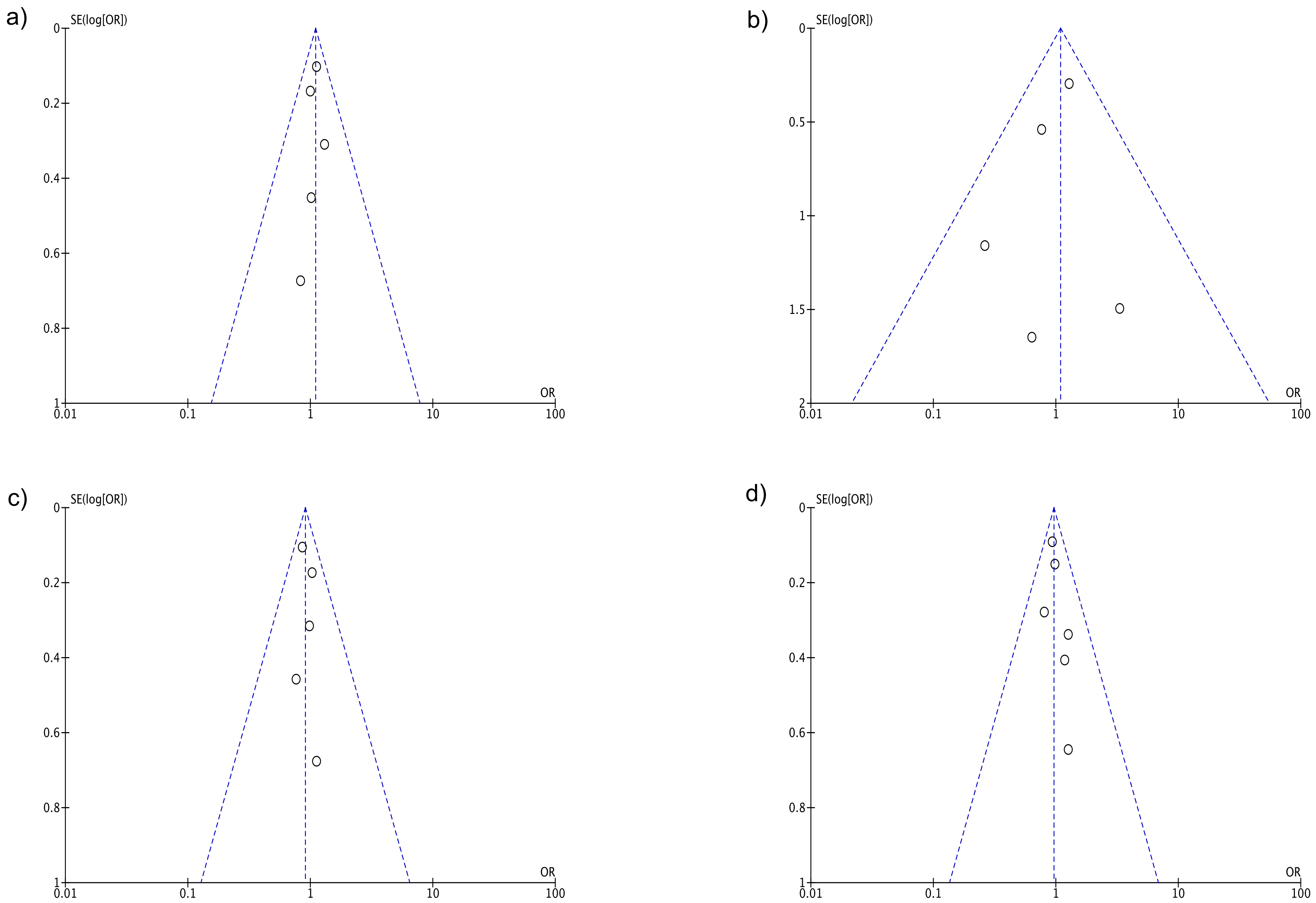

Supplementary Figure 7: Funnel plot of rs1043994 and ischemic stroke risk, under (a) dominant, (b) recessive, (c) over-dominant and (d) allelic models.

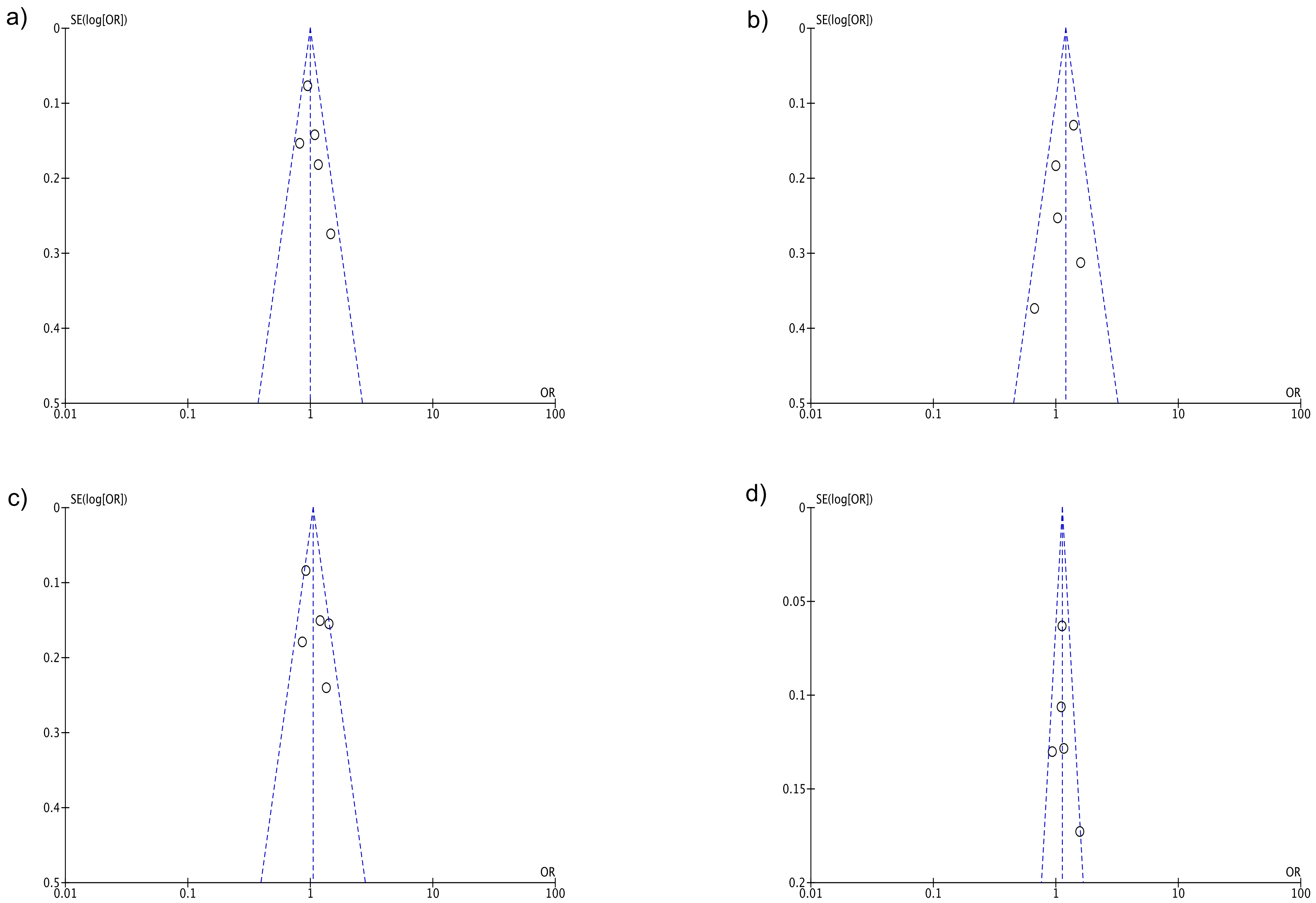

Supplementary Figure 8: Funnel plot of rs1044009 and ischemic stroke risk, under (a) dominant, (b) recessive, (c) over-dominant and (d) allelic models.

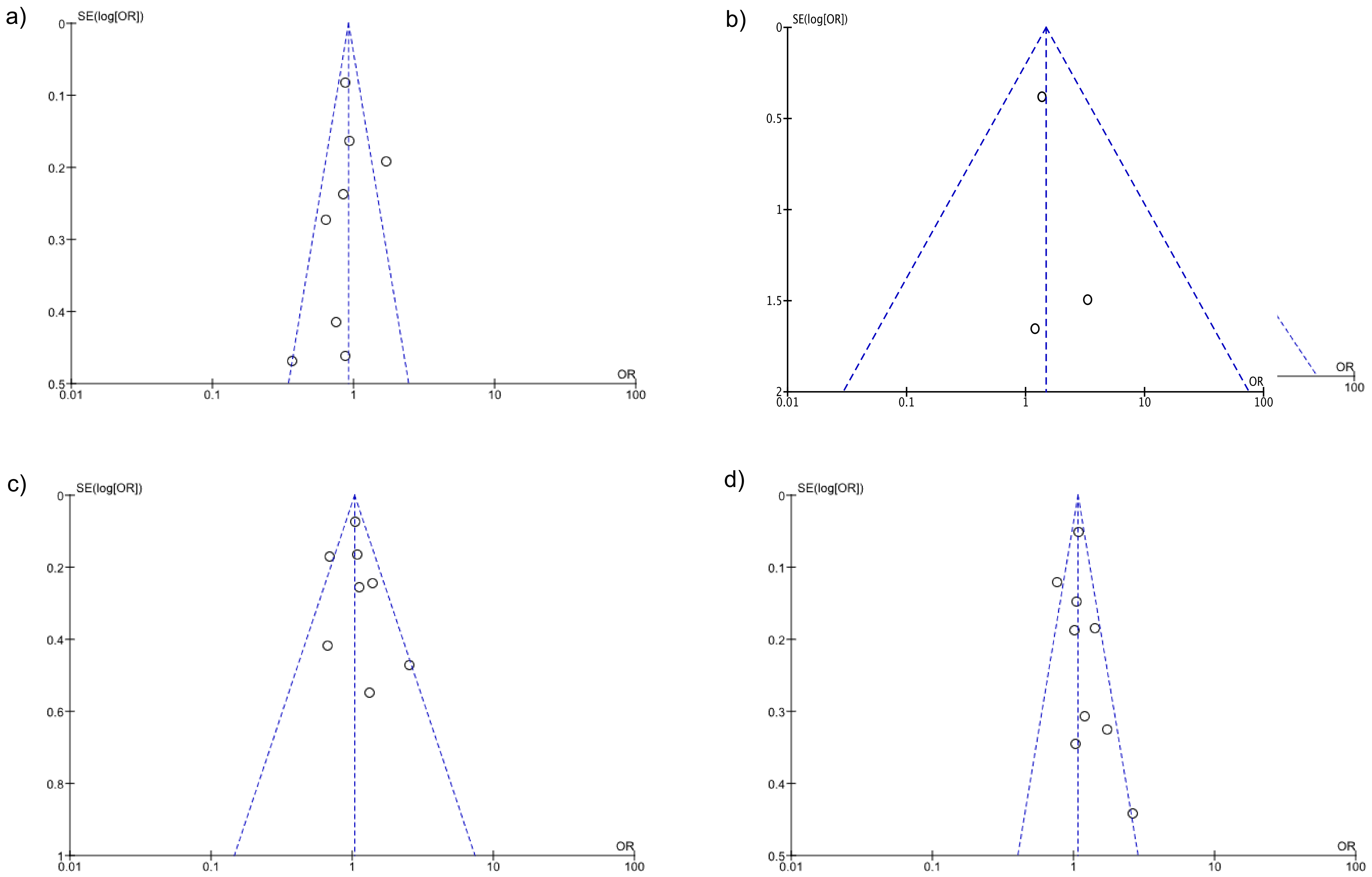

Supplementary Figure 9: Funnel plot of rs3815188 and ischemic stroke risk, under (a) dominant, (b) recessive (c) over-dominant and (d) allelic models.

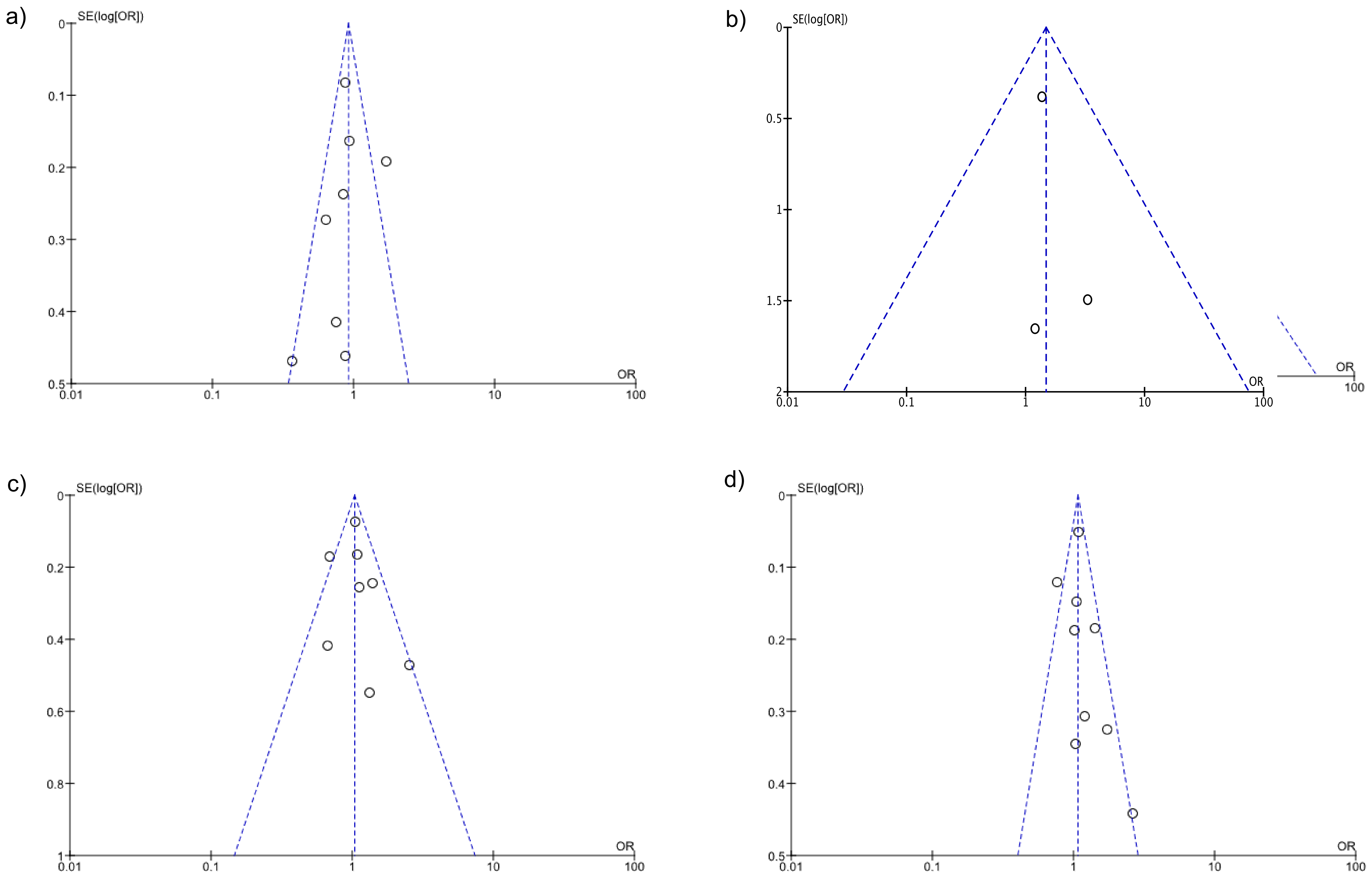

Supplementary Figure 9: Funnel plot of rs3815188 and ischemic stroke risk, under (a) dominant, (b) recessive (c) over-dominant and (d) allelic models.

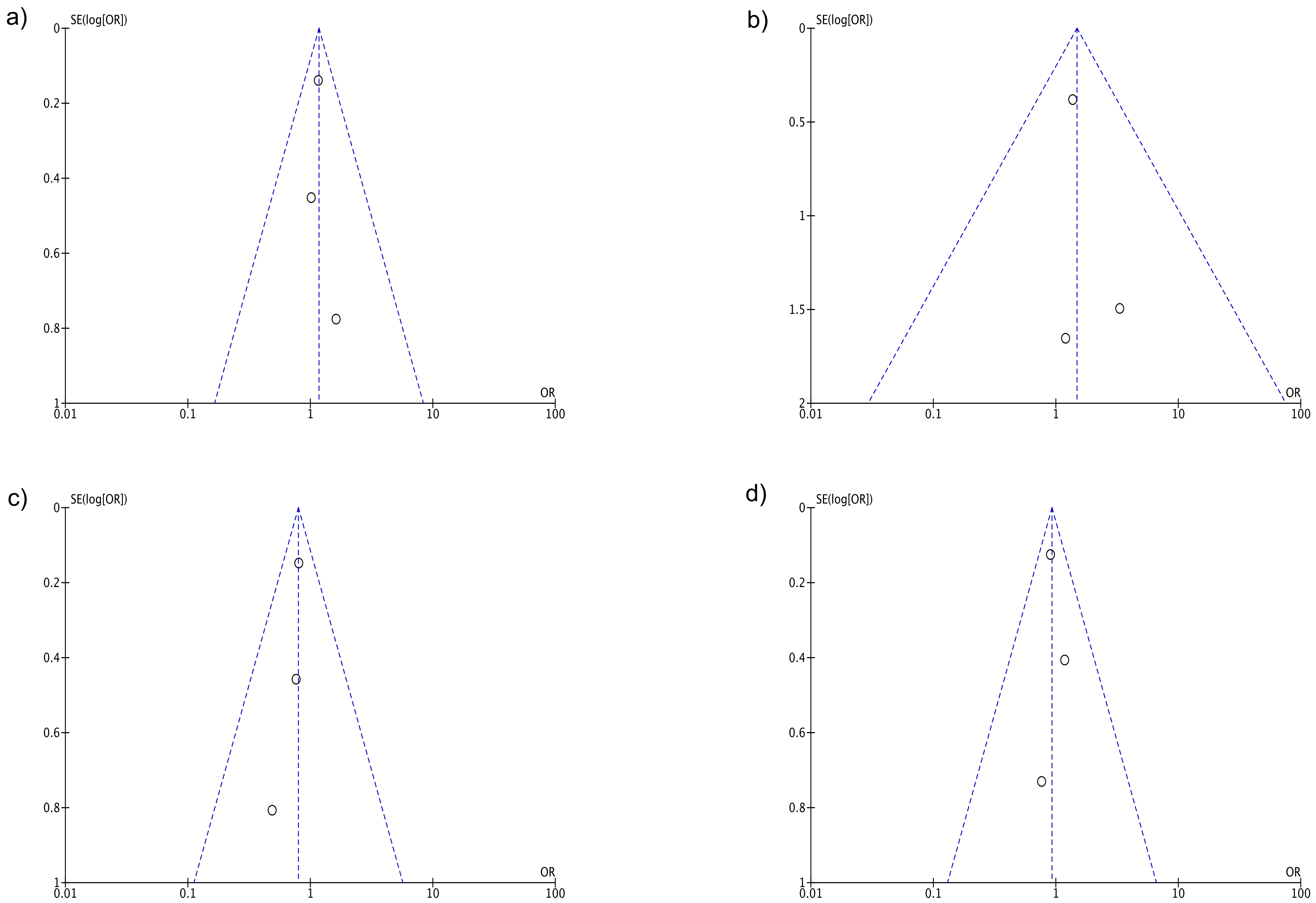

Supplementary Figure 10: Funnel plot of rs1043994 and lacunar stroke risk, under (a) dominant, (b) recessive, (c) over-dominant and (d) allelic models.

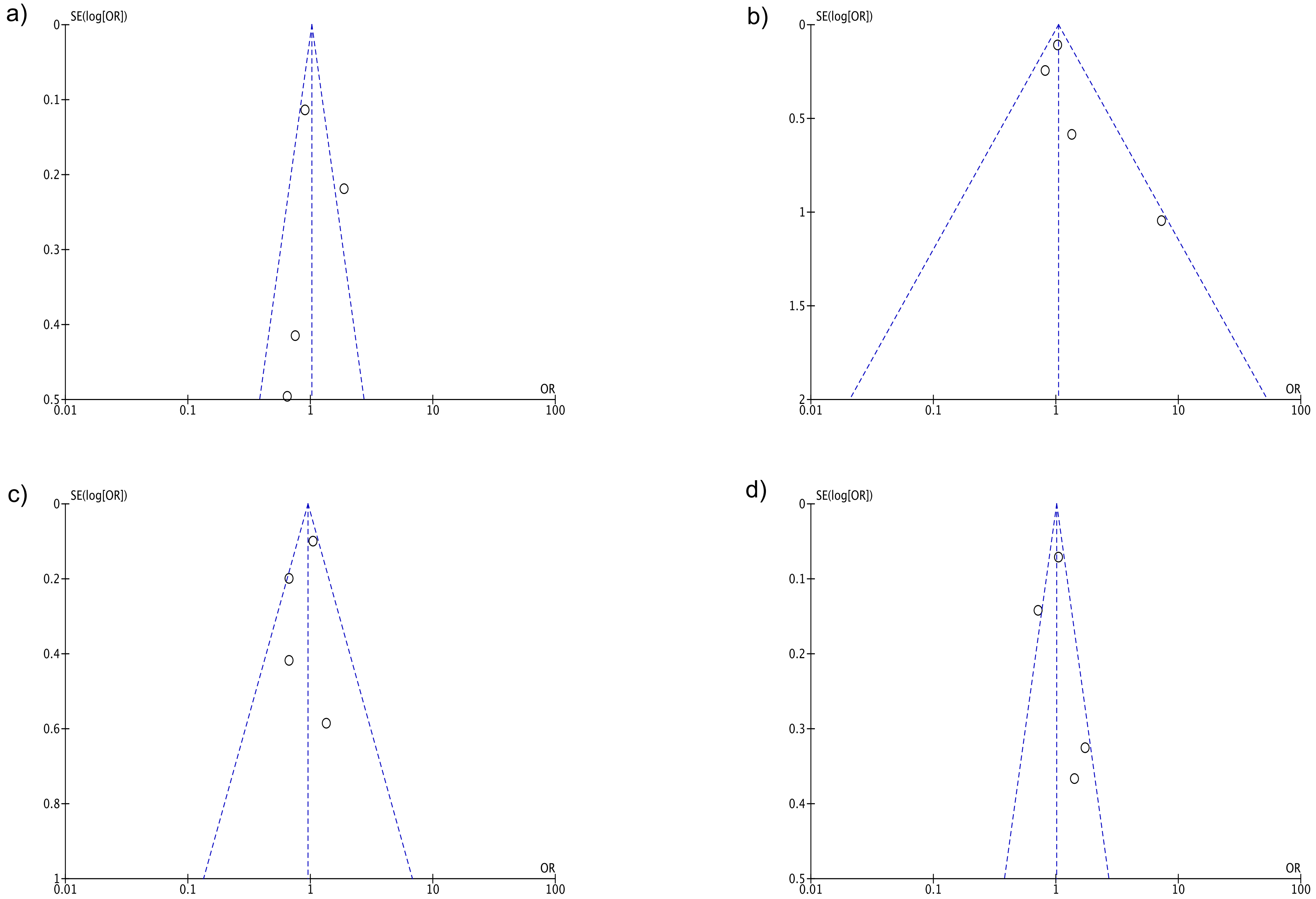

Supplementary Figure 11: Funnel plot of rs3815188 and lacunar stroke risk, under (a) dominant, (b) recessive, (c) over-dominant and (d) allelic models.

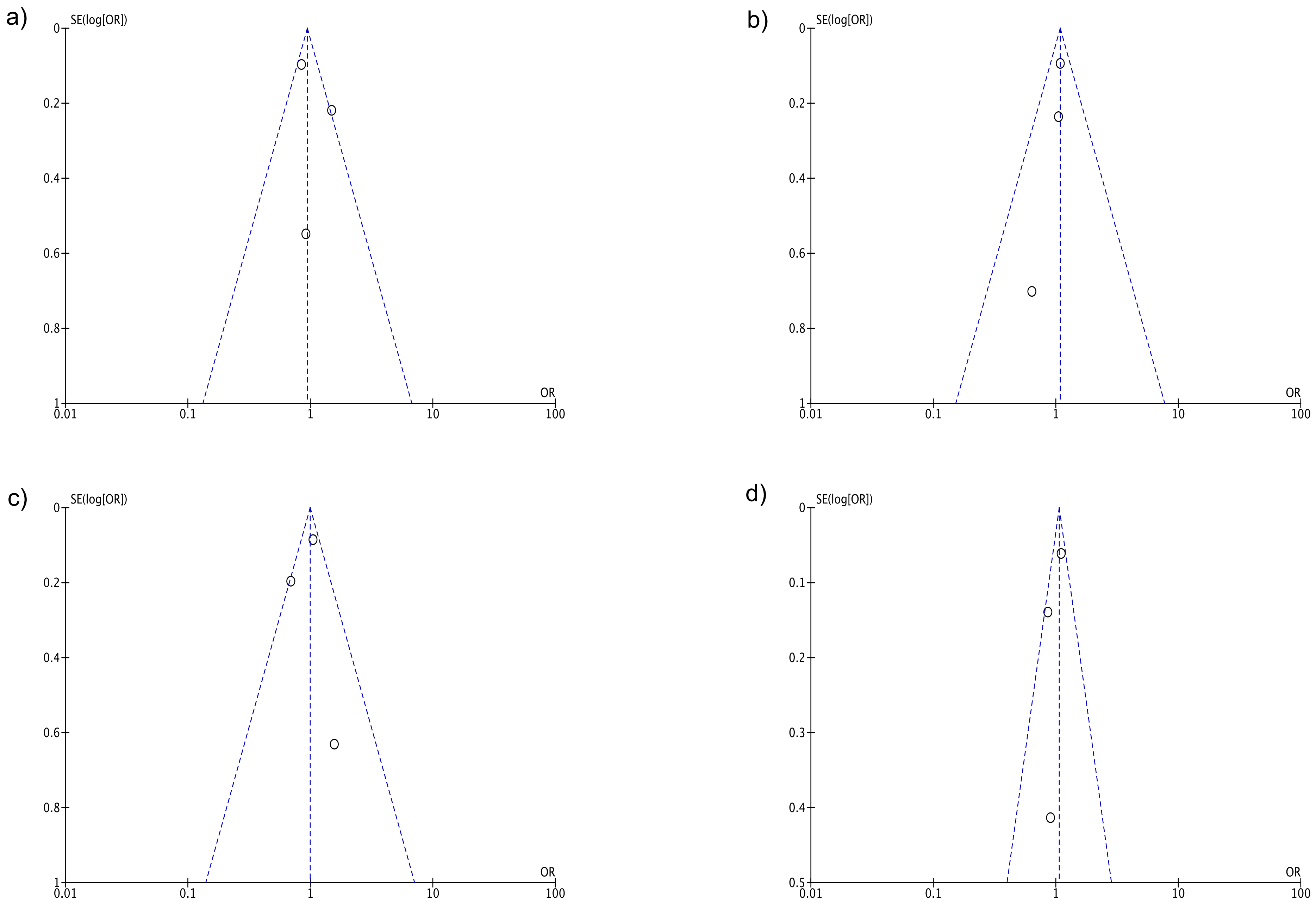

Supplementary Figure 12: Funnel plot of rss3815188 and atherothrombotic stroke risk, under (a) dominant, (b) recessive, (c) over-dominant and (d) allelic models.
