# Supplementary material for: Association of NOTCH3 Gene Polymorphisms with Ischemic Stroke and Its Subtypes: A Meta-Analysis
Source: Medicina (Kaunas). 2019 Jul 8;55(7):351. doi: 10.3390/medicina55070351 (PMC6681102; doi:10.3390/medicina55070351)
Supplement: Supplementary file 1 [file medicina-55-00351-s001.zip › Supplementary Table 1.docx]

**Supplementary Table 1** Genotype of *NOTCH3* rs1043994, rs1044009 and rs3815188 polymorphisms

1. rs1044009 and ischemic stroke

| **Author** | **Year** | **Cases** | | |  | **Controls** | | |  |
| --- | --- | --- | --- | --- | --- | --- | --- | --- | --- |
|  |  | **GG** | **Gg** | **gg** | **N** | **GG** | **Gg** | **gg** | **N** |
| Ito, D | 2002 | 87 | 93 | 31 | 211 | 119 | 151 | 45 | 315 |
| Liu, J | 2009 | 822 | 321 | 130 | 1273 | 1149 | 468 | 132 | 1749 |
| Ross, O | 2013a | 246 | 157 | 14 | 417 | 183 | 95 | 16 | 294 |
| Ross, O | 2013b | 47 | 72 | 35 | 154 | 28 | 48 | 19 | 95 |
| Zhu, C | 2016 | 93 | 113 | 54 | 260 | 242 | 233 | 125 | 600 |

1. rs3815188 and ischemic stroke

| **Author** | **Year** | **Cases** | | |  | **Controls** | | |  |
| --- | --- | --- | --- | --- | --- | --- | --- | --- | --- |
|  |  | **GG** | **Gg** | **gg** | **N** | **GG** | **Gg** | **gg** | **N** |
| Ghoreishizadeh, A | 2018 | 47 | 17 | 1 | 65 | 57 | 8 | 0 | 65 |
| Li, Y | 2013 | 45 | 39 | 21 | 105 | 15 | 14 | 1 | 30 |
| Liu, J | 2009 | 323 | 560 | 390 | 1273 | 490 | 748 | 511 | 1749 |
| Mizuno, T | 2002 | 59 | 30 | 22 | 111 | 13 | 5 | 5 | 23 |
| Ross, O | 2013a | 329 | 119 | 3 | 451 | 254 | 85 | 3 | 342 |
| Ross, O | 2013b | 86 | 68 | 11 | 165 | 72 | 43 | 13 | 128 |
| Xu, Y | 2015 | 159 | 197 | 89 | 445 | 49 | 107 | 44 | 200 |
| Yuan, X | 2016 | 37 | 76 | 21 | 134 | 43 | 62 | 10 | 115 |

1. rs3815188 and atherothrombotic stroke

| **Author** | **Year** | **Cases** | | |  | **Controls** | | |  |
| --- | --- | --- | --- | --- | --- | --- | --- | --- | --- |
|  |  | **GG** | **Gg** | **gg** | **N** | **GG** | **Gg** | **gg** | **N** |
| Liu, J | 2009 | 191 | 337 | 238 | 766 | 490 | 748 | 511 | 1749 |
| Mizuno, T | 2002 | 18 | 10 | 5 | 33 | 13 | 5 | 5 | 23 |
| Xu, Y | 2015 | 70 | 95 | 49 | 214 | 49 | 107 | 44 | 200 |

1. rs3815188 and lacunar stroke

| **Author** | **Year** | **Cases** | | |  | **Controls** | | |  |
| --- | --- | --- | --- | --- | --- | --- | --- | --- | --- |
|  |  | **GG** | **Gg** | **gg** | **N** | **GG** | **Gg** | **gg** | **N** |
| Li, Y | 2013 | 45 | 39 | 21 | 105 | 15 | 14 | 1 | 30 |
| Liu, J | 2009 | 132 | 223 | 152 | 507 | 490 | 748 | 511 | 1749 |
| Mizuno, T | 2002 | 27 | 16 | 16 | 59 | 13 | 5 | 5 | 23 |
| Xu, Y | 2015 | 77 | 88 | 38 | 203 | 49 | 107 | 44 | 200 |

1. rs 1043994 and lacunar stroke

| **Author** | **Year** | **Cases** | | |  | **Controls** | | |  |
| --- | --- | --- | --- | --- | --- | --- | --- | --- | --- |
|  |  | **GG** | **Gg** | **gg** | **N** | **GG** | **Gg** | **gg** | **N** |
| Li, Y | 2013 | 74 | 26 | 5 | 30 | 21 | 9 | 0 | 0 |
| Liu, J | 2009 | 977 | 175 | 23 | 1499 | 1221 | 255 | 23 | 0 |
| Mizuno, T | 2002 | 94 | 16 | 1 | 23 | 20 | 3 | 0 | 0 |
| Ross, O | 2013a | 345 | 97 | 7 | 347 | 267 | 73 | 7 | 0 |
| Ross, O | 2013b | 139 | 27 | 1 | 131 | 106 | 22 | 3 | 0 |
| Wang, T | 2000 | - | - | - | 70 | - | - | - | 117 |

- missing data in the primary study

N represents total number of cases and controls respectively.
